# Electrochemical Properties of a Rhodium(III) Mono-Terpyridyl Complex and Use as a Catalyst for Light-Driven Hydrogen Evolution in Water

**DOI:** 10.3390/molecules27196614

**Published:** 2022-10-05

**Authors:** Fakourou Camara, Thomas Gavaggio, Baptiste Dautreppe, Jérôme Chauvin, Jacques Pécaut, Dmitry Aldakov, Marie-Noëlle Collomb, Jérôme Fortage

**Affiliations:** 1 DCM, CNRS, Université Grenoble Alpes, 38000 Grenoble, France; 2 SyMMES, IRIG, CEA, CNRS, Université Grenoble Alpes, 38000 Grenoble, France

**Keywords:** rhodium complex, molecular electrochemistry, photocatalysis, hydrogen evolution, homogeneous catalysis

## Abstract

Molecular hydrogen (H_2_) is considered one of the most promising fuels to decarbonize the industrial and transportation sectors, and its photocatalytic production from molecular catalysts is a research field that is still abounding. The search for new molecular catalysts for H_2_ production with simple and easily synthesized ligands is still ongoing, and the terpyridine ligand with its particular electronic and coordination properties, is a good candidate to design new catalysts meeting these requirements. Herein, we have isolated the new mono-terpyridyl rhodium complex, [Rh^III^(tpy)(CH_3_CN)Cl_2_](CF_3_SO_3_) (**Rh-tpy**), and shown that it can act as a catalyst for the light-induced proton reduction into H_2_ in water in the presence of the [Ru(bpy)_3_]Cl_2_ (**Ru**) photosensitizer and ascorbate as sacrificial electron donor. Under photocatalytic conditions, in acetate buffer at pH 4.5 with 0.1 M of ascorbate and 530 μM of **Ru**, the **Rh-tpy** catalyst produces H_2_ with turnover number versus catalyst (TON_Cat_*) of 300 at a Rh concentration of 10 μM, and up to 1000 at a concentration of 1 μM. The photocatalytic performance of **Ru**/**Rh-tpy**/HA^–^/H_2_A has been also compared with that obtained with the bis-dimethyl-bipyridyl complex [Rh^III^(dmbpy)_2_Cl_2_]^+^ (**Rh2**) as a catalyst in the same experimental conditions. The investigation of the electrochemical properties of **Rh-tpy** in DMF solvent reveals that the two-electrons reduced state of the complex, the square-planar [Rh^I^(tpy)Cl] (**Rh^I^-tpy**), is quantitatively electrogenerated by bulk electrolysis. This complex is stable for hours under an inert atmosphere owing to the π-acceptor property of the terpyridine ligand that stabilizes the low oxidation states of the rhodium, making this catalyst less prone to degrade during photocatalysis. The π-acceptor property of terpyridine also confers to the **Rh-tpy** catalyst a moderately negative reduction potential (*E*p_c_(Rh^III^/Rh^I^) = −0.83 V vs. SCE in DMF), making possible its reduction by the reduced state of **Ru**, [Ru^II^(bpy)(bpy^•−^)]^+^ (**Ru**^−^) (*E*_1/2_(Ru^II^/Ru^−^) = −1.50 V vs. SCE) generated by a reductive quenching of the **Ru** excited state (*Ru) by ascorbate during photocatalysis. A Stern–Volmer plot and transient absorption spectroscopy confirmed that the first step of the photocatalytic process is the reductive quenching of *Ru by ascorbate. The resulting reduced **Ru** species (**Ru**^−^) were then able to activate the **Rh^III^-tpy** H_2_-evolving catalyst by reduction generating **Rh^I^-tpy**, which can react with a proton on a sub-nanosecond time scale to form a **Rh^III^(H)-tpy** hydride, the key intermediate for H_2_ evolution.

## 1. Introduction

**Scheme 1:**
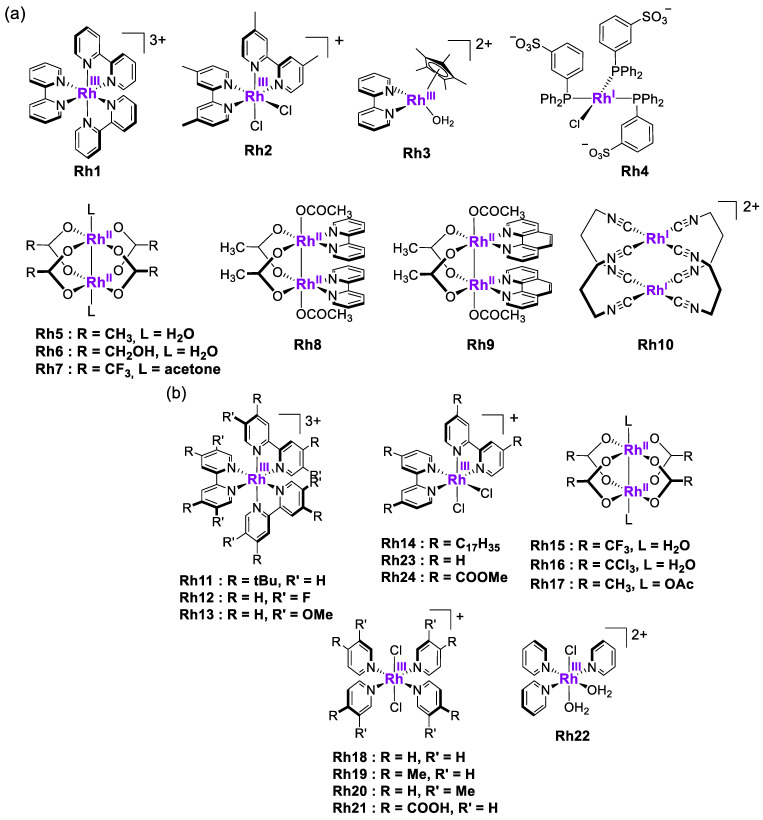
Rhodium catalysts previously reported for light-driven H_2_ production in water (**a**) and in hydro-organic solvent (**b**).

Molecular hydrogen (H_2_) is considered as one of the best alternatives to fossil fuels to speed up the transition towards a carbon-neutral future [1]. However, 96% of hydrogen is produced from fossil fuels via a variety of processes, such as the steam reforming of methane, generating carbon dioxide at the same time. Therefore, to reach carbon neutrality, hydrogen should be cleanly produced using a renewable energy, such as sunlight, with water as a proton source. The pioneer work of Lehn and Sauvage demonstrated in the late 1970s that green hydrogen could be produced by the photo-induced reduction of protons via homogeneous photocatalytic systems [2,3]. These systems are usually composed of a sacrificial electron donor (SD) providing electrons to the system, a H_2_-evolving catalyst (Cat), and a photosensitizer (PS) promoting the photo-induced electron transfers between the three compounds. For forty years, many photocatalytic systems have been investigated and numerous molecular catalysts have been employed, the latter being based on noble metal (Pd, Pt, Rh) or earth-abundant metal complexes (Co, Ni, Fe, Mo) [4,5]. Among the latter, the catalytic properties of rhodium complexes have been rarely explored in aqueous solutions (**Rh1–10**) [3,6,7,8,9,10,11,12,13,14] but more largely in hydro-organic solvent (**Rh11–24**) [10,15,16,17,18,19,20,21,22,23,24,25,26,27,28,29,30,31,32,33] (Scheme 1), although the use of water is a prerequisite for water-splitting applications [34]. Rhodium catalysts active in water for H_2_ production have employed a small variety of ligands, such as bipyridyl (**Rh1–3**, **Rh8–9**) [3,8,9,11,12,13], cyclopentadienyl (**Rh3**) [9], diphenylphosphinobenzene-sulphonate (**Rh4**) [6,7], acetate (**Rh5–9**) [8,10], and diisocyanopropane (**Rh10**) [8], under the form of mono- or bi-metallic complexes (Scheme 1a). The H_2_-evolution with such rhodium catalysts generally proceeds via the transient formation of rhodium hydride species [34,35,36] generated upon their reduction in presence of protons. Among these Rh catalysts, only **Rh2**, **Rh3**, and **Rh4** exhibited turnover numbers per catalyst (TON_Cat_) superior to 100 in the presence of a ruthenium or iridium photosensitizer and ascorbate as SD in water, and TON_Cat_ for the others catalysts do not exceed 10 [34]. In addition, when the **Rh2** bis-bipyridyl catalyst is covalently linked to two Ru photosensitizers, the performance of photocatalytic system SD/PS-Cat is clearly improved due to the faster kinetics of the electron transfer between PS and Cat [12]. In this context, exploring rhodium complexes with other polypyridyl ligands is important for identifying new efficient and robust H_2_-evolving catalysts.

**Scheme 2:**
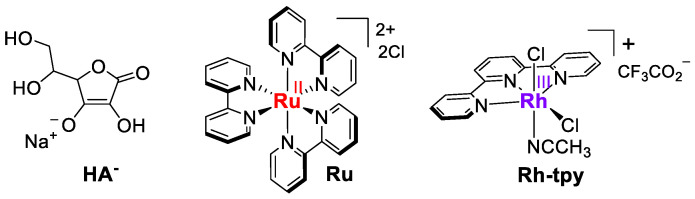
Structures of the photosensitizer (**Ru**), the H_2_-evolving catalyst (**Rh-tpy**), and the sacrificial electron donor (HA^−^) used in this work.

Herein, we report on the synthesis and structural characterization of a new rhodium mono-terpyridyl complex, namely [Rh^III^(tpy)(CH_3_CN)Cl_2_](CF_3_SO_3_) (tpy = 2,2′;6′,2″-terpyridine) (denoted **Rh-tpy**, Scheme 2). We also investigated the electrochemical properties of this complex in organic solvent and the electrochemical generation and UV-visible spectroscopic characterization of the corresponding two-electron Rh^I^ reduced species. The catalytic activity of **Rh-tpy** towards the light-driven H_2_ production was tested in purely aqueous solution, in association with [Ru(bpy)_3_]^2+^ (**Ru**) as photosensitizer and ascorbate (HA^−^) as sacrificial electron donor. In 2012, the group of Ogo investigated the capacity of the chemically synthetized rhodium(I) terpyridyl [Rh^I^(tpy)(CH_3_CN)](CF_3_SO_3_) complex to form a rhodium(III) monohydride species in the presence of protons in CH_3_CN, [Rh^III^(H)(tpy)(CH_3_CN)_2_](CF_3_SO_3_)_2_, the latter being unstable and gradually transforming into a stable dinuclear Rh^II^ complex [Rh_2_^II^(tpy)_2_(CH_3_CN)_4_](CF_3_SO_3_)_4_, with the generation of H_2_ by reductive elimination [37]. In this study, we demonstrate that the **Rh-tpy** can act as a H_2_-evolving catalyst under photocatalytic conditions in acidic water (pH 4.0–4.5) with an efficiency competing with the benchmark rhodium(III) bis-dimethyl-bipyridyl complex [Rh^III^(dmbpy)_2_Cl_2_]^+^ (**Rh2**) (Scheme 1) for a low catalyst concentration of 1 μM, this latter being among the most efficient rhodium catalysts in water [11,12,13]. Finally, the first photo-induced steps involved in the H_2_ production with the molecular **Ru**/**Rh-tpy**/HA^−^/H_2_A system are investigated by means of time-resolved emission and absorption spectroscopies.

## 2. Results and Discussion

### 2.1. Synthesis and Crystal Structure of the [Rh^III^(tpy)(CH_3_CN)Cl_2_](CF_3_SO_3_) Complex

**Scheme 3:**
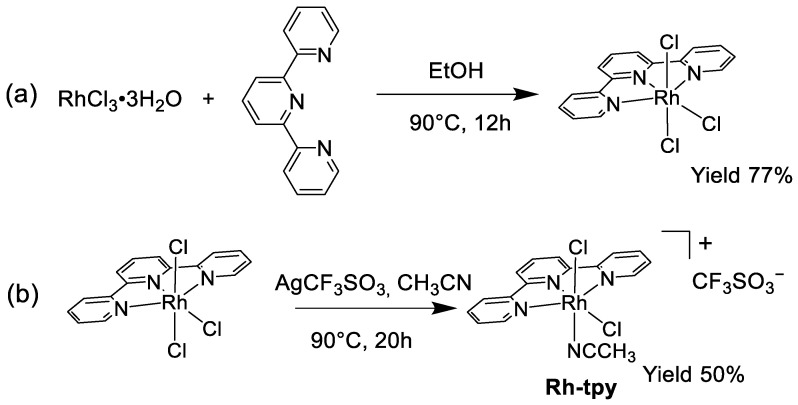
Synthesis of the [Rh^III^(tpy)Cl_3_] (**a**) and [Rh^III^(tpy)(CH_3_CN)Cl_2_](CF_3_SO_3_) (**b**) (**Rh-tpy**) Complexes.

The synthesis of the **Rh-tpy** complex was inspired from that of the [Rh^III^(tpy)(OH)(H_2_O)_2_](NO_3_)_2_ complex isolated by Ogo (Scheme 3) [38]. First, the [Rh^III^(tpy)Cl_3_] complex is generated by reacting RhCl_3_•3H_2_O with one equivalent of terpyridine in ethanol at 90 °C [39]. Then, in order to solubilize the rhodium complex in water for the light-driven H_2_ production experiments, a chloride ligand in axial position was exchanged by an acetonitrile molecule by reacting the tris-chloro complex with AgCF_3_SO_3_ in acetonitrile at 90 °C, leading to the formation of the [Rh^III^(tpy)(CH_3_CN)Cl_2_](CF_3_SO_3_) complex (**Rh-tpy**) with a yield of 50%. The water solubility of **Rh-tpy** is also favored by the presence of the triflate counter-anion. 

**Figure 1:**
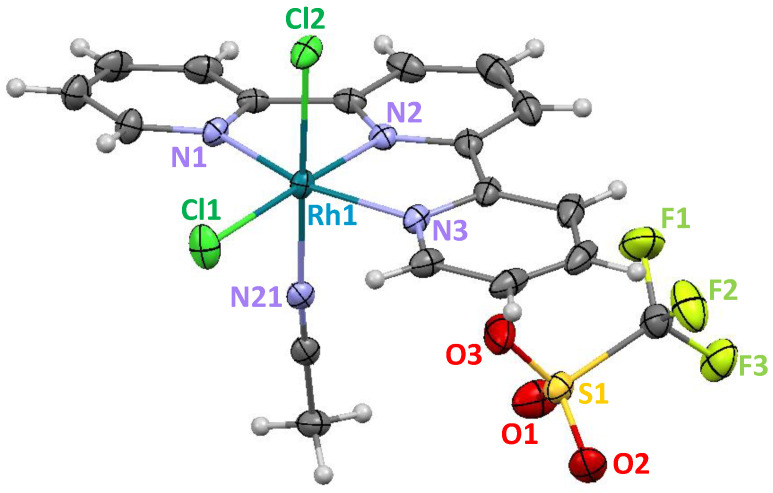
ORTEP diagram of [Rh^III^(tpy)(CH_3_CN)Cl_2_](CF_3_SO_3_) (**Rh-tpy**). Thermal ellipsoids are drawn at the 50% probability level. Selected distances (Å) and angles (º): Rh1-N1 = 2.0258(14) Å, Rh1-N2 = 1.9386(14) Å, Rh1-N3 = 2.0417(14) Å, Rh1-N21 = 2.0220(14) Å, Rh1-Cl1 = 2.3530(5) Å, Rh1-Cl2 = 2.3067(4) Å, N1-Rh1-N3 = 162.03(6)º, N2-Rh1-N1 = 81.07(6)º, N2-Rh1-N3 = 81.00(6)º, N2-Rh1-N21 = 87.90(6)º.

Single crystals of **Rh-tpy** suitable for X-ray crystallography were obtained by diffusion of diethyl ether into an acetonitrile solution of the rhodium complex (Figure 1, Tables S1–S4). **Rh-tpy** crystallizes in the *P*1 space group and displays a distorted octahedral geometry around the rhodium atom. The three nitrogen atoms of the terpyridine and one chloride ligand are in the equatorial plane, while the nitrogen atom of CH_3_CN and one chloride ligand are in an axial position. The terpyridine ligand is quasi-planar, with low torsion angles between the central pyridine of the terpyridine and the other two pyridines (3.90° between pyridines with N(1) and N(2) and 1.70° between pyridines with N(2) and N(3)). The nitrogen atoms of the terpyridine, the chloride atom, and the rhodium form a slightly distorted square plane, with N(1)-Rh-N(2) and N(2)-Rh-N(3) angles of 81.07(6)° and 81.00(6)°, respectively, which are slightly smaller than the ideal value of 90° for a perfect square plane geometry. This distortion is induced by the terpyridine, which imposes 5-atom rings comprising the rhodium, two nitrogen atoms (N(1)/N(2) or N(2)/N(3)), and two terpyridine carbon atoms. The distance between rhodium and the central pyridine nitrogen (N_central_) of the terpyridine (N(2)) is shorter (1.9386(14) Å) than that between rhodium and the two distal nitrogen (N_distal_) of the terpyridine (2.0258(14) and 2.0417(14) Å for Rh-N(1) and Rh-N(3), respectively). The rhodium atom is almost equidistant from the two chloride atoms (2.3530(5) vs. 2.3067(4) Å, respectively, for Rh-Cl(1) and Rh-Cl(2)) and the distance between the nitrogen of acetonitrile ligand and the rhodium atom (2.0220(14) Å for Rh-N(21)) is very similar to those between the rhodium and the terpyridine nitrogen atoms (Rh-N_tpy_). The distances and angles between the Rh center and the terpyridine ligand in **Rh-tpy** are very similar to those found in the [Rh^III^(tpy)(OH)(OH_2_)_2_](NO_3_)_2_ [38]. Slightly shorter valence angles were found in the dinuclear [Rh_2_^II^(tpy)_2_(CH_3_CN)_4_](CF_3_SO_3_)_4_ complex where the two Rh centers are linked by a metal-metal bond, as a consequence of Rh^III^-N_tpy_ lengths being slightly longer than those of **Rh-tpy**. Regarding the low-valent [Rh^I^(tpy)(CH_3_CN)](CF_3_SO_3_) complex, which adopts a discrete structure of four [Rh^I^(tpy)(CH_3_CN)]^+^ units stacked via tpy-tpy interaction at the solid state, this complex displays slightly shorter valence angles and Rh^III^-N_tpy_ bond distances than those found in **Rh-tpy** [37].

### 2.2. Spectroelectrochemical Properties of the [Rh^III^(tpy)(CH_3_CN)Cl_2_](CF_3_SO_3_) Complex (Rh-tpy) in N,N-Dimethylformamide (DMF)

**Figure 2:**
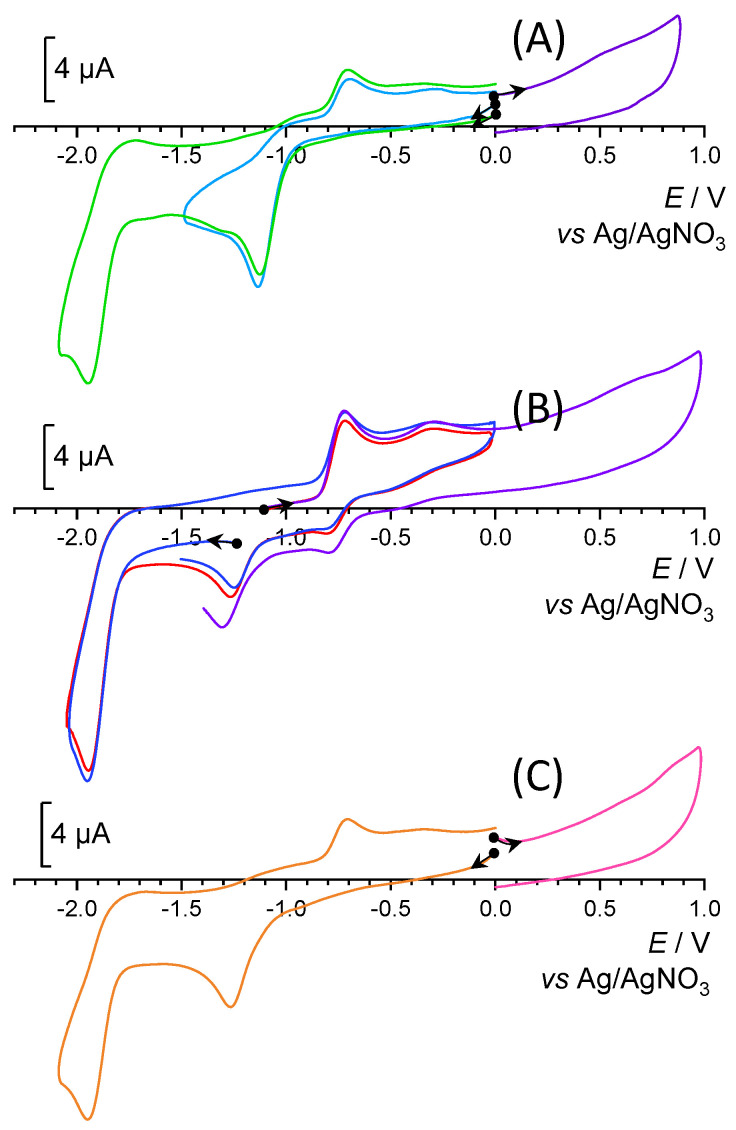
Cyclic voltammograms at a glassy carbon electrode (Ø = 3 mm, ν = 100 mV s^−1^) of (**A**) 0.5 mM solution of **Rh-tpy** in DMF, 0.1 M [Bu_4_N]ClO_4_, (**B**) after a two-electron exhaustive reduction at –1.25 V vs. Ag/AgNO_3_ leading to the formation of the Rh(I) species, and (**C**) after a back exhaustive oxidation at 0 V.

The redox properties of **Rh-tpy** were investigated in DMF because of the wider electrochemical window of this solvent compared to water, facilitating the observation of the reduced state of the rhodium complex (Figure 2 and Table S5). By analogy with the previously reported electrochemical behavior of the rhodium bis-bipyridyl [11,12,13,36,40] and bis-terpyridyl [41] complexes in organic solvent, the irreversible redox process at *E*p_a_ = −1.13 V vs. Ag/AgNO_3_ observed on the cyclic voltammogram of **Rh-tpy** is attributed to the two-electron reduction of the metal center (Rh^III^ → Rh^I^), while the poorly reversible process located at a lower potential (*E*p_c_ = −1.95 V) is ascribed to the one-electron reduction of the terpyridine ligand (Figure 2A). The irreversibility of the Rh^III^/Rh^I^ system is the result of the release of the two axial ligands upon reduction to form a Rh^I^ species with a square-planar geometry. On the reverse scan, the irreversible oxidation peak observed at *E*p_a_ = −0.70 V, followed by another less defined at ca. −0.3 V, is related to the reoxidation of the metal center (Rh^I^ → Rh^III^) along with the recoordination of two exogeneous ligands, which can be the solvent (DMF), CH_3_CN, and/or a chloride ion.

**Figure 3:**
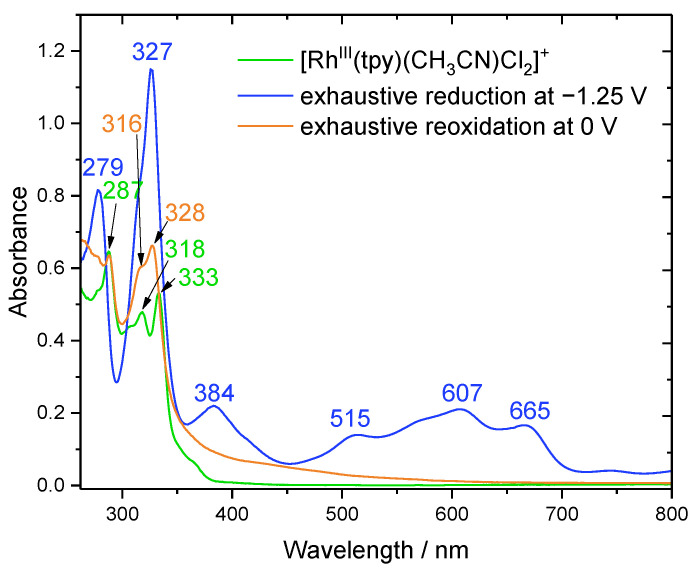
UV-vis absorption spectra of 0.5 mM solution of **Rh-tpy** in DMF, 0.1 M [Bu_4_N]ClO_4_ before (green trace) and after a two-electron exhaustive reduction at −1.25 V vs. Ag/AgNO_3_ leading to the formation of the [Rh^I^(tpy)Cl] (blue trace), followed by a back exhaustive oxidation at 0 V (orange trace). Optical path length was 1 mm.

In order to further characterize and evaluate the stability of the reduced Rh^I^ state of **Rh-tpy**, the initial species involved in the catalytic reduction of protons to H_2_, an electrolysis was performed at −1.25 V. In accordance with the generation of a Rh^I^ species, the exhaustive electrolysis required the exchange of two electrons per initial Rh^III^ complex. The formation of this species was attested by the UV-Visible absorption spectrum (Figure 3) and cyclic voltammograms (Figure 2B) of the resulting electrolyzed solution. The initial pale yellow **Rh-tpy** solution displays mainly intense absorption bands below 350 nm. After complete reduction, the deep blue solution obtained from Rh^I^ exhibits two strong absorption bands at 279 and 327 nm, and four less intense absorption bands in the visible region at 384, 515, 607, and 665 nm (Figure 3). Very similar absorption spectra were reported by the group of Hartl in ethanol for the square-planar rhodium(I) terpyridyl halide complexes, [Rh^I^(tpy)(X)] (X = Cl or Br), chemically synthesized from [Rh(X)(COD)]_2_ precursors (X = Cl or Br; COD = cycloocta-1,5-diene) under inert atmosphere [42]. The similarity of the absorption spectra strongly suggests the [Rh^I^(tpy)Cl] structure for the electrogenerated Rh^I^ species as a result of the release of one CH_3_CN and one chloride ligand. 

Regarding the cyclic voltammograms of the Rh^I^ solution, the initial irreversible reduction peak (Rh^III^ → Rh^I^) has fully disappeared and only the redox process centered on the tpy ligand is now present on the cyclic voltammogram, with an intensity similar to that of the initial solution of [Rh^III^(tpy)(MeCN)Cl_2_]^+^ (Figure 2A,B), attesting to the stability of the Rh^I^ species under inert atmosphere. In addition, the irreversible oxidation peaks (Rh^I^ → Rh^III^) observed by scanning from −1.1 to 0 V are accompanied, on the reverse scan, by the appearance of a Rh^III^ → Rh^I^ reduction peak in accordance with the regeneration of a Rh^III^ species. Besides, the UV-vis. spectrum and the cyclic voltammograms of the electrolyzed solution do not show any changes after several hours when the latter is stored under inert atmosphere free of oxygen (glove box), showing the excellent stability of the Rh^I^ terpyridyl complex. By contrast, under similar experimental conditions, solutions of [Rh^I^(Rbpy)_2_]^+^ electrogenerated from rhodium(III) bis-bipyridyl complexes [Rh^III^(Rbpy)_2_Cl_2_]^+^ (R = dimethyl or ditertio-butyl) are less stable and start to decompose after 1.5 h [36]. This is likely due to the higher π-acceptor capacity of terpyridine compared to that of bipyridine, which discharges the excess of electron density of Rh^I^ to the ligand, thus stabilizing the low oxidation states of the metal [42]. The rigid and planar geometry of the tpy ligand can also contribute to the stability of the square planar Rh^I^ species. A back exhaustive electrolysis at *E* = 0 V restores a Rh^III^ complex. However, the two-electron reduction of the metal center (Rh^III^ → Rh^I^) is now located at a slightly more negative potential (*E*p_a_ = −1.27 V vs. Ag/AgNO_3_) compared to the initial solution of **Rh-tpy**, suggesting a slightly different coordination sphere for the Rh^III^ center, which might be the substitution of the initial CH_3_CN axial ligand by DMF. Indeed, the two chloride ligands remain coordinated to the Rh^III^ center, as judged by the absence of the characteristic electroactivity of free chlorides (irreversible oxidation peak at ca. 0.7 V vs. Ag/AgNO_3_) in the positive potential region from **Rh-tpy** [36].

This electrochemical study shows that the activation of the **Rh-tpy** complex for photo-induced H_2_ production is possible using **Ru** as photosensitizer. Indeed, **Rh-tpy** can be reduced either by the reduced [Ru^II^(bpy)(bpy^•−^)]^+^ (denoted **Ru**^−^) or by the excited states of **Ru** (denoted *Ru^II^), since both reactions are favored from a thermodynamic point of view. Considering the reduction potential of **Rh-tpy** (*E*p_c_ (Rh^III^/Rh^I^) = −1.13 V vs. Ag/AgNO_3_ (−0.83 V vs. SCE)) in DMF, and the redox potential of **Ru** at reduced **Ru**^−^ and excited *Ru^II^ states in water (*E*_1/2_ (Ru^II^/Ru^−^) = −1.50 V and *E*_1/2_ (Ru^III^/*Ru^II^) = −1.07 V vs. SCE [3], see Table S5), the driving forces (ΔG_0_) are clearly exergonic with values of −0.67 and −0.24 eV, respectively. Photophysical experiments (see below) allowed for deciphering the photocatalytic mechanism occurring with the system **Ru**/**Rh-tpy**/HA^−^/H_2_A.

### 2.3. Photocatalytic Hydrogen Production

The catalytic activity of **Rh-tpy** for the light-driven production of H_2_ was evaluated in the presence of the **Ru** photosensitizer and ascorbate as a sacrificial electron donor in deaerated aqueous solutions (5 mL) at 298 K under visible light irradiation (400–700 nm). The light-driven evolution of H_2_ was determined by gas chromatography in the course of the photocatalysis, and these data were used to calculate the TON (turnover number) and initial TOF values (turnover frequency) per molecule of catalyst (respectively denoted as TON_Cat_ and TOF_Cat_), which characterize the performance of the photocatalytic system (Table S7). 

**Figure 4:**
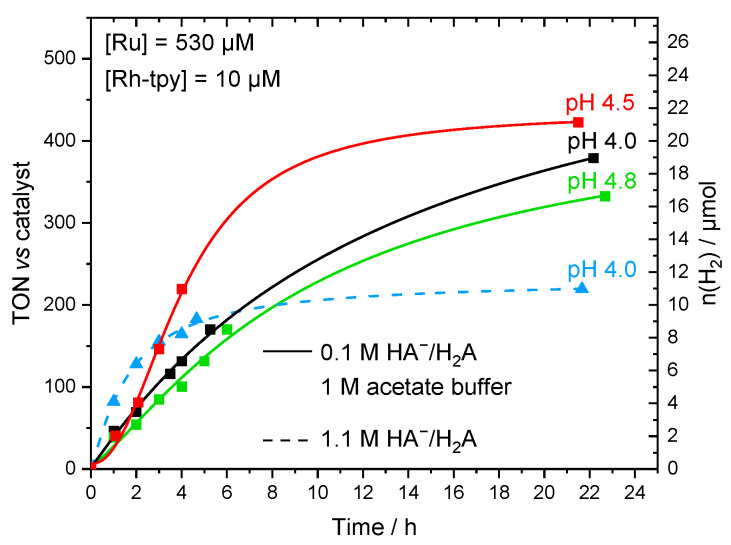
Photocatalytic hydrogen production (TON_Cat_) as a function of time under 400–700 nm irradiation from a deaerated aqueous solution (5 mL) of **Ru** (530 μM) and **Rh-tpy** (10 μM) containing an acetate buffer (1 M) and NaHA/H_2_A (0.1 M) at different pHs (4.0; 4.5; 4.8) (solid trace) or only a buffer of NaHA/H_2_A (1.1 M) at pH 4.0 (dotted trace).

**Figure 5:**
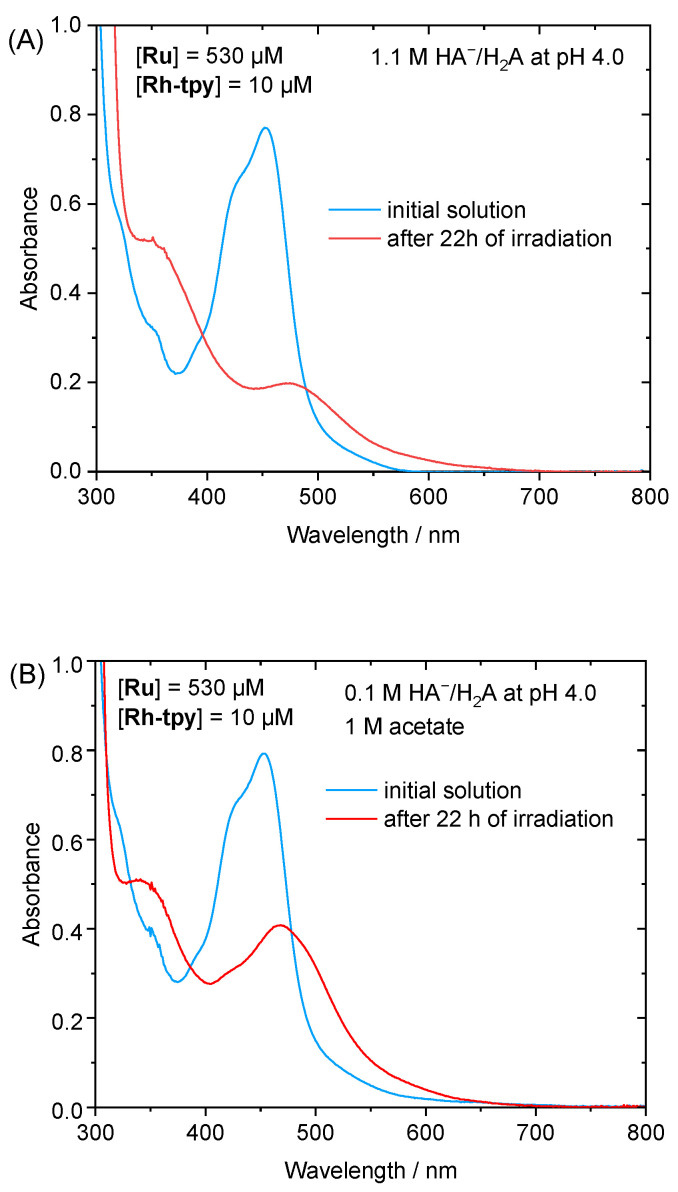
UV-vis absorption spectra of (**A**) **Ru** (530 μM) in aqueous solution containing NaHA/H_2_A (1.1 M) at pH 4.0 in presence of **Rh-tpy** (10 μM), (**B**) **Ru** (530 μM) in acetate buffer (1 M) at pH 4.0 containing NaHA/H_2_A (0.1 M) in presence of **Rh-tpy** (10 μM), initial solutions and after about 22 h of irradiation. Optical path = 1 mm.

The catalytic activity of **Rh-tpy** was first investigated in a HA^−^/H_2_A buffer (total concentration 1.1 M, see Table S6) at pH 4.0 with 530 μM of PS, similar to our previous experiments with **Rh2** [11,12] and cobalt catalysts [43,44,45,46,47]. In these conditions, the **Ru**/**Rh-tpy**/HA^−^/H_2_A system displays 220 TON_Cat_ after 22 h of irradiation with a catalyst concentration of 10 μM (Figure 4). However, the stability of the photocatalytic system did not exceed 7–8 h and about 90% of the total amount of H_2_ was achieved after 6 h. The UV-vis. absorption spectrum of the photocatalytic solution recorded at the end of the photocatalysis indicates the complete transformation of the **Ru** PS into the bis-bipyridyl Ru(II) derivative, [Ru(bpy)_2_(X)_2_]^n+^, with the disappearance of the initial absorption band at 450 nm of **Ru** and the appearance of a new shoulder at 473 nm with a lower intensity (Figure 5A). This is due to the well-known poor stability of the reduced state **Ru^−^** in acidic water, generated upon the reductive quenching of *Ru by HA^−^ (see below), which easily undergoes a ligand substitution by solvent molecules and/or anions such as ascorbate [48,49]. In view of slowing down the degradation of **Ru** and thus improving the stability of the photocatalytic system, a lower HA^−^/H_2_A concentration of 0.1 M was used. To keep the pH constant during the photocatalytic experiment, the aqueous solution was buffered with an acetate buffer (1 M) at pH 4.0. Such conditions have been already employed for several photocatalytic systems for H_2_ production involving cobalt catalysts [48,50,51,52,53]. In acetate buffer containing 0.1 M of HA^−^/H_2_A at pH 4.0, the **Ru**/**Rh-tpy** photocatalytic system is clearly more stable, since the H_2_ production is still effective after 22 h of irradiation. At this stage, the TON_Cat_ value has reached 380, which is almost twice as high as the TON_Cat_ value obtained in 1.1 M HA^−^/H_2_A buffer, and the **Ru** PS is less degraded at the end of photocatalysis (Figure 5B). However, when the concentration of the sacrificial electron donor is divided by 10, the initial TOF_Cat_ value is lower than that obtained in 1.1 M HA^−^/H_2_A (38 vs. 95 TOF_Cat_, respectively, see Table S7). The pH was further optimized using the acetate buffer with 0.1 M HA^−^/H_2_A medium. The highest TON_Cat_ value of 423 was obtained at pH 4.5 after 22 h of irradiation. A further increase of the pH to 4.8 leads to a decrease of the TON_Cat_ value (Figure 4). Basically, the pH at which photocatalysis is optimal is governed by a delicate balance between the reactivity of the catalyst (generation of the Rh^III^ hydride species) and the efficiency of the photoinduced electron transfer process (concentration of both protonated and non-protonated form of the SD) [34,54]. More acidic conditions make the protonation of the catalyst, i.e., the H_2_-evolution catalysis, faster, but the generation of the reductant **Ru**^−^ is slowed down because the concentration of the ascorbate electron donor is smaller. In other words, in less acidic conditions, the higher concentration of ascorbate makes the quenching or regeneration of PS more efficient, but the low concentration in protons decreases the H_2_-evolving activity of the catalyst.

**Figure 6:**
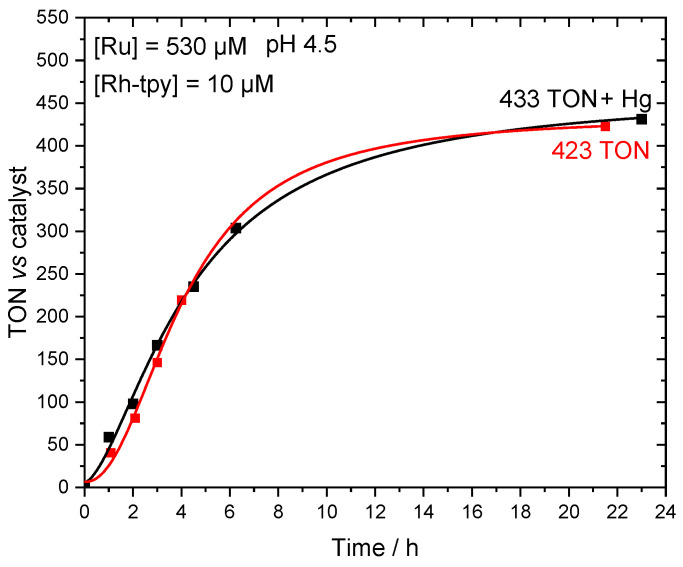
Photocatalytic hydrogen production (TON_Cat_) as a function of time under 400–700 nm irradiation from deaerated 1 M acetate aqueous buffer (5 mL) at pH 4.5 containing NaHA/H_2_A (0.1 M), in the presence of **Ru** (530 μM) and **Rh-tpy** (10 μM) with (black trace) and without (red trace) one drop of mercury.

In order to rule out the formation of Rh^0^ colloids from **Rh-tpy** during the photocatalysis, an experiment was carried out with 530 μM of **Ru**, 10 μM of **Rh-tpy** in acetate buffer (0.1 M) containing HA^−^/H_2_A (0.1 M) at pH 4.5, in the presence of an excess of mercury (Figure 6). Rh^0^ colloids are known to catalyze the reduction of protons to H_2_ and can be deactivated by generating an amalgam with mercury [55,56]. The absence of effect on the photocatalytic behavior of the **Ru**/**Rh-tpy**/HA^−^/H_2_A system due to the Hg addition confirms that no colloidal rhodium is formed during photocatalysis and the molecular **Rh-tpy** catalyst is involved in the H_2_ evolution.

**Figure 7:**
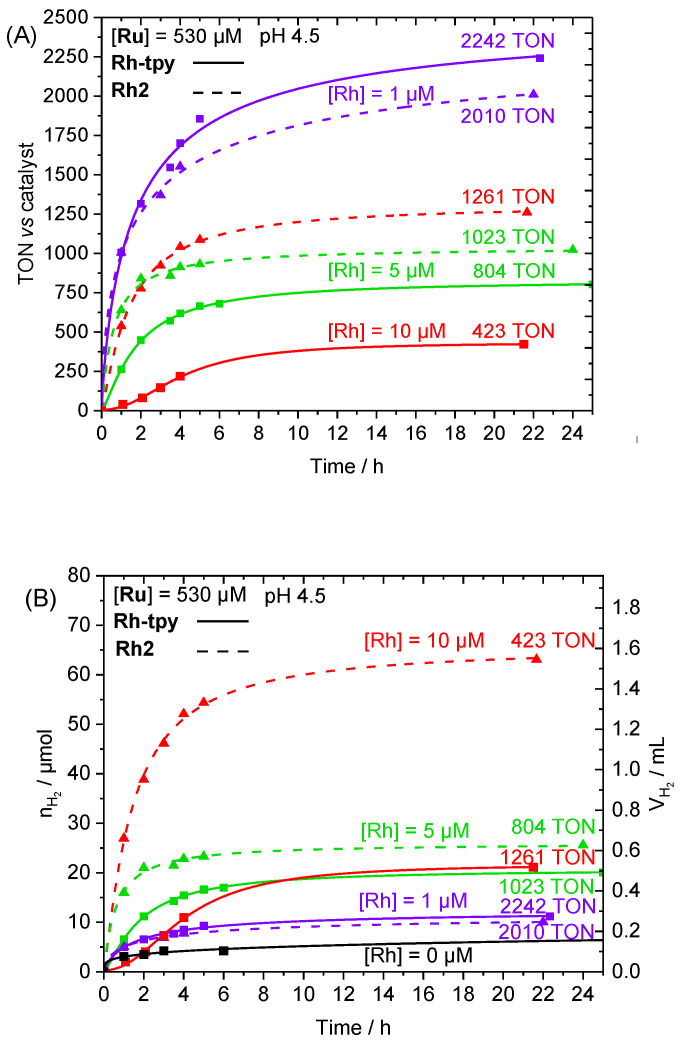
Photocatalytic hydrogen production as a function of time under 400–700 nm irradiation from a deaerated 1 M acetate aqueous buffer (5 mL) at pH 4.5 containing NaHA/H_2_A (0.1 M), in the presence of **Ru** (530 μM) and **Rh-tpy** at 10, 5, and 1 μM: (**A**) TON_Cat_ and (**B**) n_H2_ and V_H2_.

The photocatalytic performances of the **Ru**/**Rh-tpy**/HA^−^/H_2_A system were also investigated at lower concentrations of **Rh-tpy** in acetate buffer at pH 4.5 by keeping the concentration of **Ru** constant in view to promote the reduction of the catalyst (Figure 7). At 5 and 1 μM, the TON_Cat_ values increase drastically to reach 804 and 2242, respectively, after 22–25 h of irradiation. This strong increase of TON_Cat_ values when the PS/Cat ratio increases has been previously observed in many photocatalytic molecular systems using rhodium [11,15] or cobalt catalysts [45,46,47,48,49,53,57,58,59,60,61,62,63]. However, these values do not take into account the amount of H_2_ that comes only from the **Ru** photosensitizer in the absence of a catalyst. Indeed, while control experiments in the absence of **Ru** or HA^−^ do not produce any H_2_, some H_2_ is generated from solutions containing only **Ru** and HA^−^/H_2_A (Table S7, Figure 7B). This amount is not negligible, as it represents about 30% of the H_2_ produced for the 10 and 5 μM concentrations and more than half (55%) for the lowest concentration of 1 μM. Consequently, the actual TON_Cat_ values (denoted as TON_Cat_*), calculated by subtracting the amount of H_2_ stemming from **Ru**, are thus of 300, 558, and 1012 at 10, 5, and 1 μM, respectively (Table S7). 

**Figure 8:**
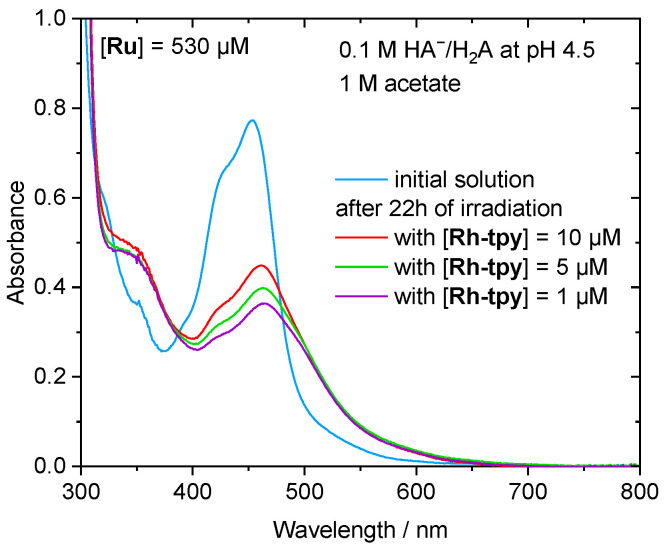
UV-vis absorption spectra of **Ru** (530 μM) in acetate buffer (1 M) at pH 4.5 and NaHA/H_2_A (0.1 M), initial solution and after about 22 h of irradiation with **Rh-tpy** at various concentrations (10, 5, and 1 μM). Optical path = 1 mm.

UV-vis absorption spectra recorded at the end of the photocatalysis show that the Ru PS is partially transformed in Ru-bis-bipyridine species and that this transformation is more pronounced as the concentration of the catalyst is low (Figure 8) [53]. The degradation of the PS is most probably the main limiting factor for the H_2_ production after 22 h [54].

The photocatalytic performance of **Ru**/**Rh-tpy**/HA^−^/H_2_A has also been compared with that obtained with **Rh2** as catalyst in the same experimental conditions (Table S7). In combination with a molecular photosensitizer, the **Rh2** complex is among the most efficient H_2_ evolving catalysts based on rhodium that operate in water [11,12,15,34]. At pH 4.5 in 1 M acetate buffer containing 0.1 M of HA^−^/H_2_A, the **Rh2** catalyst appears significantly more active than **Rh-tpy** for the higher catalyst concentration of 10 μM, with corrected TON_Cat_* of 1140 vs. 300 for **Rh-tpy**. However, in contrast to what was observed for **Rh-tpy** and, in a previous reported study for **Rh2** in the 1.1 M HA^−^/H_2_A buffer [11], if the catalyst concentration is decreased, the TON_Cat_* also decreases to about 780 at 5 μM and 770 at 1 μM (Figure 7). Thus, **Rh-tpy** is clearly less efficient than **Rh2** at the higher catalyst concentrations of 10 and 5 μM (TON_Cat_* of 300 and 558 for **Rh-tpy** vs. 1140 and 778 for **Rh2**), but challenges **Rh2** in more diluted conditions (for 1 μM, TON_Cat_* of 1012 for **Rh-tpy** vs. 772 for **Rh2**). In fact, the higher stability of the Rh^I^ species for **Rh-tpy** compared to that of **Rh2**, as revealed in organic solvent, could explain the photocatalytic performance of **Ru**/**Rh-tpy**/HA^−^/H_2_A competing with that of **Ru**/**Rh2**/HA^−^/H_2_A at very low concentrations of catalyst. Therefore, the use of tridentate tpy ligand compared to bidentate bpy ligands leads to a less active but potentially more stable H_2_-evolving catalyst. However, the instability of **Ru** PS does not allow for comparing the long-term stability of **Rh-tpy** and **Rh2** catalysts. A more stable PS in water such as the triazatriangulenium TATA^+^ organic dye should be employed, but the reduction potential of this PS, which is less negative than that of **Ru [53]**, will not allow for an efficient reduction of the Rh catalysts.

We also summarized the performances of the various three-components photocatalytic systems for H_2_ production in homogeneous solution in Tables S8 and S9, involving a rhodium complex (**Rh1–24**, Scheme 1) as a H_2_-evolving catalyst previously reported in view to compare the efficiency of these systems with those of **Rh2** and **Rh-tpy** as catalysts. Tables S8 and S9 summarize the performances in hydro-organic (**Rh11–24**, Scheme 1b) and in purely aqueous (**Rh1–10**, Scheme 1a) solution, respectively. The structures of the various photosensitizers employed in these photocatalytic systems are gathered in Scheme S1. In aqueous organic media, PSs mainly rely on heteroleptic cyclometaled iridium(III) complexes of the type [Ir(C^N)_2_(N^N)]^+^ (C^N = phenylpyridine ligand and N^N = bipyridine ligand) with various substituents, although there are a few examples employing copper. In purely aqueous solution, most of the systems rely on **Ru**. Reliable comparison of the efficiency of the different systems is not obvious, since in addition to the fact that the experimental conditions are carried out in different experimental set-up, TON_Cat_ values are strongly dependent on the PS and Cat concentrations, the PS/Cat ratio, the nature of the solvent and of sacrificial electron donor, and the long-term stability of the PS.

Among the Rh catalysts employed in water (**Rh1–10**, Scheme 1a), only **Rh2**, **Rh3,** and **Rh4** exhibited turnover numbers per catalyst (TON_Cat_) higher than 100 in the presence of a ruthenium or iridium photosensitizer and ascorbate as SD [11]; TON_Cat_ for the others catalysts, **Rh5–10**, do not exceed 10 [3,8] (Table S9). However, these later systems used significantly higher catalyst concentration ranging from 50 μM to 1.56 mM, and the catalyst concentration is generally higher than that of the PS, resulting in lower TON_Cat_. Concerning the Wilkinson catalyst **Rh4**, although up to 2000 TONs_Cat_ were reported [6,7], further studies by our group have shown that this catalyst is much less efficient than **Rh2** [11].

Significantly higher TON_Cat_ were obtained in hydro-organic media. The highest TON_Cat_ in such media, from 2362 up to 9886, were obtained by the group of Kataoka with the **Rh5**, **Rh15–16** and **Rh11** catalysts in association with **Ir1**, **Ir2**, and **Ir7** as PSs in THF/H_2_O [28,29] (Table S8). In these examples, the concentration ranges are similar to our studies, i.e., 500 μM for the PS and 5 μM for the catalyst. Interestingly, in the other studies combining **Ir3–5** (100 μM) and **Rh1** or **Rh11–14** (100 μM) of Bernhard [15], **Ir1** (50 μM) and **Rh8–9** or **Rh17** (50 μM) of Wang [10] and **Ir2** (926 μM) and **Rh18–21** (926 μM) of Kozlova [32,33], for which the Ir/Cat ratio is close to 1/1, TONs_Cat_ are comprised between 15 and 150, and reach 380 in one case. 

These examples show that even with quite similar families of catalysts (for instance, **Rh5–9** [8] and **Rh5**, **Rh15–16 [28,29]**) and PS, the TON_Cat_ values can be very different depending on the experimental conditions. The most relevant comparisons are therefore those made under similar experimental conditions. We can thus compare the efficiency of our systems with rhodium with those of the cobalt(III) tetraazamacrocyclic [Co^III^(CR14)Cl_2_]^+^ (**Co**) complex, which is one of the most efficient H_2_-evolving catalyst in acidic water [43,45,47,53]. The catalytic performances of this complex have been recorded under similar experimental conditions with 500 μM **Ru** and 0.1 M of HA^−^/H_2_A in 1 M acetate buffer at pH 4.5 for catalyst concentrations of 10 and 5 μM (Table S7) [53]. TON_Cat_* of 1086 and 1822 were obtained for **Co** compared to 300 and 558 for **Rh-tpy** for 10 and 5 μM, respectively. At both catalysts’ concentrations, **Rh-tpy** is thus much less efficient than **Co**, with more than three times less hydrogen produced.

### 2.4. Mechanistic Insight for the Ru/Rh-tpy/HA^−^/H_2_A System from Photophysical Measurements

**Scheme 4:**
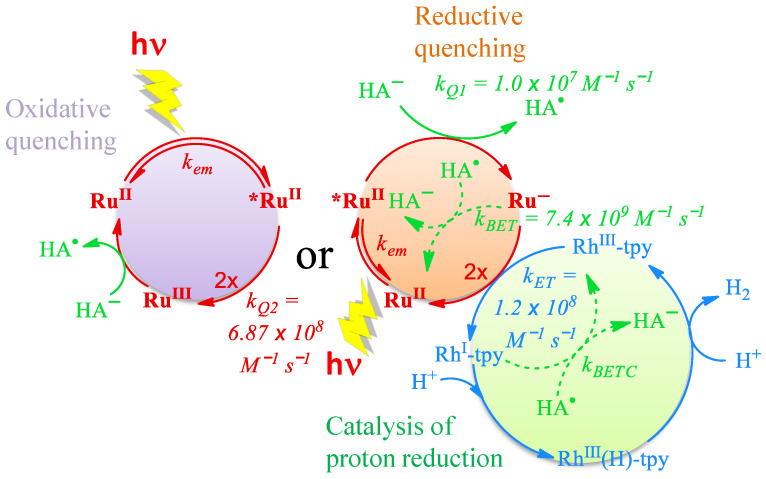
Proposed catalytic mechanism for the light-driven H_2_ production with the system **Ru**/**Rh-tpy**/HA^−^/H_2_A. For reasons of simplicity, only the pathway showing the heterolytic mechanism from **Rh^III^(H)-tpy** is shown. *Ru^II^ represents the triplet excited state of Ru^II^, and Ru^−^, its one-electron reduced state.

The light-driven H_2_ production with the **Ru**/**Rh-tpy**/HA^−^/H_2_A three-component system is initiated by the generation of the excited state of the **Ru**, *Ru^II^, under light absorption (Scheme 4). *Ru could be then quenched by an electron transfer to the sacrificial electron donor (HA^−^) to generate the reduced form of PS, **Ru**^−^, and the oxidized form of ascorbate (HA^•^) (reductive quenching). **Ru**^−^ is able, in turn, to reduce the catalyst (**Rh^III^-tpy** to **Rh^I^-tpy**), reforming the ground state **Ru**. HA^•^ can lose a proton to form A^•−^, which can disproportionate generating the dehydroascorbic acid (DHA) [34,64,65], a very good electron acceptor able to withdraw electrons from **Ru**^−^ and/or **Rh^I^-tpy**. Therefore, a back electron transfer takes place from **Ru**^−^ to HA^•^ or DHA (BET process) to restore **Ru^II^** and HA^−^, or from **Rh^I^-tpy** to HA^•^ or DHA (BETC process) to regenerate **Rh^III^-tpy**.

The *Ru can also be quenched by the catalyst leading to the oxidized form of the PS, **Ru^III^**, and the reduced state of catalyst, **Rh^I^-tpy** (oxidative quenching), this process being unfavorable (see below). 

**Figure 9:**
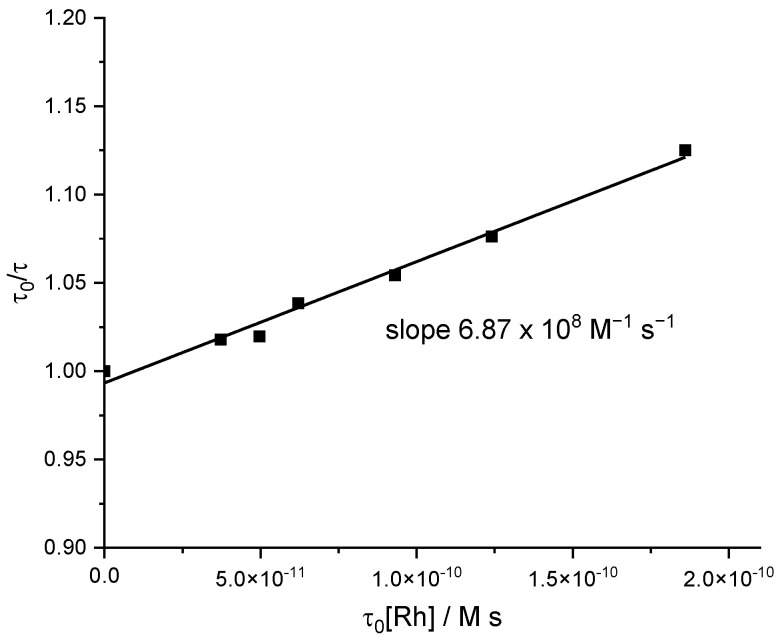
Stern–Volmer plot performed in a deaerated aqueous solution of acetate buffer (1 M) at pH 4.5 for the oxidative quenching of **Ru** (10 μM) at an excited state by **Rh-tpy** by varying its concentration (0; 0.06; 0.08; 0.1; 0.15; 0.2; 0.3 mM). The lifetime of *Ru without catalyst (τ_0_) was determined to be 620 ns in water at pH 4.5.

Photophysical measurements allow us to identify which one of these two mechanisms is the most favorable. By using a Stern–Volmer plot, a rate constant (k_Q1_) of 1.0 × 10^7^ M^−1^ s^−1^ was determined by the Schmehl’s group for the reductive quenching of the *Ru^II^ luminescence by HA^−^ in an aqueous acetate buffered at pH 4.5. In the same medium, we determined a rate constant of 6.87 × 10^8^ M^−1^ s^−1^ for the oxidative quenching (k_Q2_) of *Ru by **Rh-tpy** (Figure 9), which is about 70 times higher than that of the reductive quenching of *Ru by HA^−^. Although the oxidative quenching is kinetically more favored than the reductive quenching, the reductive pathway dominates over the oxidative one with pseudo-first-order kinetics of 1.0 × 10^6^ s^−1^ and 0.069–3.4 × 10^4^ s^−1^, respectively, considering that the HA^−^ concentration (0.1 M) is much higher than that of **Rh-tpy** (1–10 μM) under photocatalytic conditions. Noteworthily, the rate constant of the oxidative quenching of *Ru by **Rh^III^-tpy** is very similar to that previously determined by our group between *Ru and **Rh2** (3.2 × 10^8^ M^−1^ s^−1^) [11]. This similarity of rate constants could be correlated to their akin driving forces ΔG_0_ of −0.24 eV for *Ru/**Rh-tpy** and −0.28 eV for *Ru/**Rh2**, considering the reduction potentials (Rh^III^/Rh^I^) of **Rh-tpy** and **Rh2**, and the oxidation potential of *Ru (see Table S5).

Nanosecond flash photolysis experiments have also been performed to characterize the photoinduced electron transfer process occurring in the system **Ru**/**Rh-tpy**/HA^−^/H_2_A at pH 4.5. In the absence of **Rh-tpy**, the transient absorption spectra recorded after excitation at 455 nm (Figure S1) show the formation of the **Ru**^−^ species with positive absorption bands at 360 and 510 nm [66]. The formation of **Ru**^−^ occurs from an electron transfer between *Ru and HA^−^ [9,11]. **Ru**^−^ growth follows a pseudo first-order kinetics with an estimated rate constant of 4.3 × 10^6^ s^−1^ (τ = 235 ns, Figure S2). The decay of the transient absorption trace at 510 nm, due to the back electron transfer (BET) between **Ru**^−^ and the oxidized forms of ascorbate (HA^•^ and DHA), can be fitted according to a second order kinetics law (Figure S3). The rate constant (k_BET_) is estimated to be 7.4 × 10^9^ M^−1^ s^−1^.

**Figure 10:**
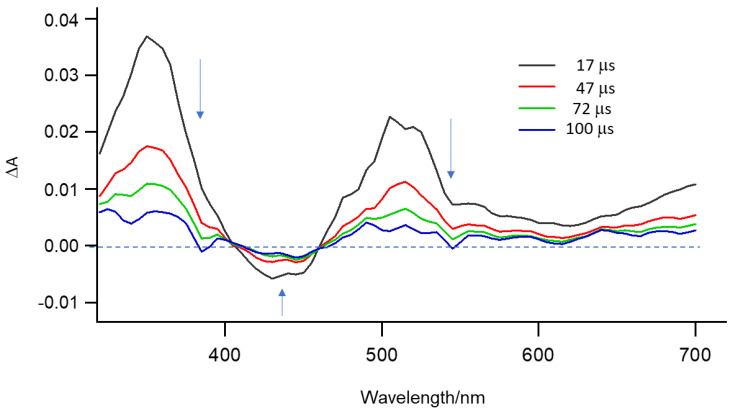
Transient absorption spectra recorded at different times after flash laser excitation (λ = 455 nm) of a deaerated aqueous solution of acetate buffer (1 M) at pH 4.5 containing **Ru** (100 μM), NaHA/H_2_A (0.1 M), and **Rh-tpy** (200 μM) (path length = 1 cm).

**Figure 11:**
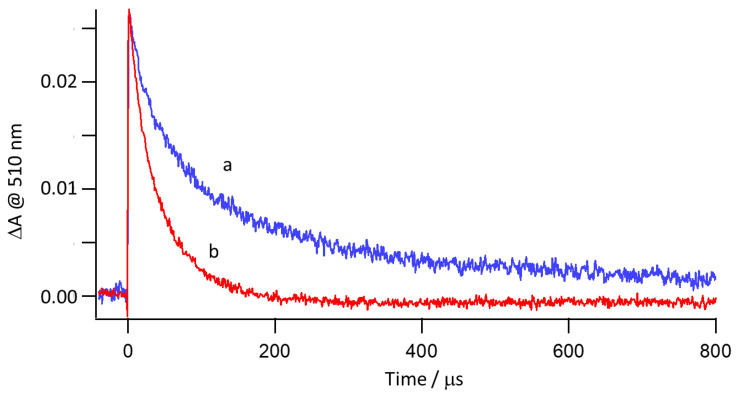
Transient absorption traces on the microsecond time scale recorded at 510 nm after laser excitation at 455 nm of a deaerated aqueous solution of acetate buffer (1 M) at pH 4.5 containing: (a) **Ru** (100 μM) and NaHA/H_2_A (0.1 M), (b) **Ru** (100 μM), NaHA/H_2_A (0.1 M), and **Rh-tpy** (200 μM).

In the presence of **Rh-tpy**, flash photolysis experiments also show the formation of the **Ru**^−^ species, evidenced by an increase of absorption at 360 and 510 nm (Figure 10). The growth of the signal occurs in a similar time scale (τ = 215 ns, corresponding to a constant at 4.6 × 10^6^ s^−1^, Figure S4) to that observed without **Rh-tpy** (Figure S2). This confirms that the photocatalytic cycle is initiated by a reductive quenching of *Ru by HA^−^ and that the presence of **Rh-tpy** does not interfere with this first electron transfer process. However, the decay of the transient absorption trace at 510 nm is accelerated in the presence of **Rh-tpy** (Figure 11). This is a consequence of an electron transfer process between the transient **Ru**^−^ and **Rh^III^-tpy** leading to the initial **Ru^II^** and **Rh^I^-tpy**, which efficiently competes with the BET process between **Ru**^−^ and the oxidized ascorbate (e.g., HA^•^ and DHA).

In the presence of **Rh-tpy**, the decay of the transient **Ru**^−^ species is best fitted by a pseudo-first-order kinetics law, leading to a rate constant for the ET process of 2.4 × 10^4^ s^−1^ (Figure S5). Considering the concentration of the catalyst in solution (200 μM), this would correspond to a bimolecular rate constant (k_ET_) of 1.2 × 10^8^ M^−1^ s^−1^, which is 62 times lower than the k_BET_ value (7.4 × 10^9^ M^−1^ s^−1^). The difference between these two kinetics should contradict the faster decay of **Ru**^−^ in presence of **Rh^III^-tpy** observed in the transient absorption trace in Figure 11. Nevertheless, we have estimated the initial concentration of **Ru**^−^ (2.27 × 10^−7^ M), calculated from the concentration of *Ru^II^ (9.1 × 10^−7^ M) just after the excitation pulse (ΔA@450 nm = −0.01 and Δε = −11,000 M^−1^ cm^−1^) and considering a quantum yield of 25% for the **Ru**^−^ formation from reaction between *Ru and HA^−^ [45]. This has allowed us to determine the real rates of the ET and BET processes (v_ET_ and v_BET_) at 5.45 × 10^−3^ and 3.81 × 10^−4^ M s^−1^, respectively (see ESI for the calculation details). In other words, under our experimental conditions, the ET process dominates over the BET process with a v_ET_/v_BET_ ratio of 14.3. Finally, although the two-electron reduced catalyst (**Rh^I^-tpy**) exhibits a large absorption band between 550 and 700 nm (Figure 3), its typical spectroscopic signature is not observed in the transient absorption spectra. This is attributed to the fast reactivity of the **Rh^I^-tpy** species (i.e., [Rh^I^(tpy)X]^n+^, X = Cl^−^ or H_2_O with n = 0 or 1, respectively) with protons, below the nanosecond time-scale, to form a **Rh^III^(H)-tpy** hydride species (i.e., [Rh^III^(H)(tpy)(H_2_O)X]^n+^, X = Cl^−^ or H_2_O with n = 1 or 2, respectively), the key intermediate to reduce protons into H_2_. Indeed, from this species, different pathways can lead to the production of H_2_ via homolytic (reaction with another **Rh^III^(H)-tpy** species to generate H_2_ and two Rh(II) species) or heterolytic (reaction with a proton to form H_2_ and a Rh(III) species) route (Scheme 4). **Rh^III^(H)-tpy** could be also further reduced by **Ru**^−^ to a Rh(II) hydride species, **Rh^II^(H)-tpy**, from which H_2_ can be released via similar homolytic and heterolytic pathways, generating Rh(I) and Rh(II) species, respectively. The group of Ogo has shown that in CH_3_CN, the hydride complex [Rh^III^(H)(tpy)(CH_3_CN)_2_]^2+^ can slowly generate H_2_ via reductive elimination leading to the Rh(II) dimer species (homolytic route) [37]. However, we cannot rule out that in acidic water, other pathways can proceed in parallel to this homolytic mechanism. For instance, according to theoretical calculations, we have shown that, for the **Rh2** catalyst in acidic water, H_2_ is preferentially released through a heterolytic mechanism from the Rh^III^(H) species and that both homo- and heterolytic mechanisms are thermodynamically favorable to generate H_2_ via the Rh^II^(H) species [13]. Furthermore, although the formation of the **Rh^III^(H)-tpy** hydride species is very fast, the catalysis of proton reduction generally remains the rate-determining step of the photocatalytic mechanism, occurring within three-component systems for photocatalytic evolution of H_2_ in homogeneous media [44,54]. 

## 3. Materials and Methods

### 3.1. Synthesis of [Rh^III^(tpy)Cl_3_]

RhCl_3_•3H_2_O (150 mg, 0.55 mmol) and 2, 2′:6′; 2″-terpyridine (129 mg, 0.55 mmol) were dissolved in absolute ethanol (24 mL). After stirring at 90 °C for 12 h, a yellow precipitate appeared to correspond to [Rh^III^(tpy)Cl_3_], which was filtered off from the hot reaction mixture and washed with diethyl ether (188 mg, yield, 77%). ^1^H NMR (DMSO-d_6_), 400 MHz, δ (ppm): 9.28 (d; J = 5.2 Hz; 2H); 8.81 (d; J = 8 Hz; 2H); 8.77 (d; J = 7.6 Hz; 2H); 8.54 (t; J = 8 Hz; 1H); 8.38 (t; J = 7.6 Hz; 2H); 7.95 (t; J = 6.4 Hz; 2H). IR (cm^−1^): 3069, 2932, 1598, 1570, 1472, 1447, 1396, 1312, 1252, 1241, 1184, 1157, 1136, 1048, 1026, 751, 668, 655. ESI-MS, positive mode *m*/*z* (%): 405.87 (59) [M − Cl]^+^, 463.83 (81) [M + Na]^+^.

### 3.2. Synthesis of [Rh^III^(tpy)(CH_3_CN)Cl_2_](CF_3_SO_3_) (Rh-tpy)

[Rh^III^(tpy)Cl_3_] (116 mg, 0.26 mmol) was added to an acetonitrile solution (232 mL) of AgCF_3_SO_3_ (209 mg, 0.81 mmol). After stirring at 90 °C for 20 h, the white precipitate of AgCl formed was filtered off from the hot reaction mixture. The resulting pale yellow filtrate was concentrated until 30 mL, yielding to a yellow precipitate of [Rh^III^(tpy)(CH_3_CN)Cl_2_](CF_3_SO_3_), which was filtered off and washed with diethyl ether to give a pale yellow powder. [Rh^III^(tpy)(CH_3_CN)Cl_2_](CF_3_SO_3_) was recrystallized by slow diffusion of diethyl ether into an acetonitrile solution of complex (80 mg, yield, 51%). ^1^H NMR (D_2_O), 400 MHz, δ (ppm): 9.06 (d; J = 5.6 Hz; 2H); 8.66 (d; J = 7.6 Hz; 2H); 8.59 (d; J = 7 Hz; 1H); 8.46 (t; J = 8 Hz; 2H); 8.02 (t; J = 7.6 Hz; 2H); 2.94 (s, 3H); IR: 3088, 2930, 2324, 1604, 1575, 1479, 1452, 1403, 1320, 1258, 1223, 1139, 1030, 773, 635. ESI-MS, positive mode *m*/*z* (%): 446.95 (100) [M − CF_3_SO_3_]^+^, 405.93 (26) [M − CF_3_SO_3_-CH_3_CN]^+^.

## 4. Conclusions

We demonstrated, for the first time, that the terpyridyl rhodium complex, [Rh^III^(tpy)(CH_3_CN)Cl_2_](CF_3_SO_3_) (**Rh-tpy**), is able to catalyze the light-induced proton reduction into H_2_ in aqueous solution in the presence of the [Ru^II^(bpy)_3_]Cl_2_ (**Ru**) photosensitizer and ascorbate as sacrificial electron donors. Under photocatalytic conditions, TON_Cat_ values of 300 and up to 1000 were obtained for H_2_ production for a **Rh-tpy** catalyst concentration at 10 and 1 μM, respectively, after subtraction of the amount of H_2_ stemming from the **Ru** only. The photocatalytic performance of **Ru**/**Rh-tpy**/HA^−^/H_2_A has also been compared with that obtained with **Rh2** as a catalyst in the same experimental conditions. It appears that **Rh-tpy** is clearly less efficient than **Rh2** at the higher catalyst concentrations of 10 and 5 μM (TON_Cat_* of 300 and 558 for **Rh-tpy** vs 1140 and 778 for **Rh2**), but challenges **Rh2** in more diluted conditions (for 1 μM, TON_Cat_* of 1012 for **Rh-tpy** vs. 772 for **Rh2**). Therefore, the use of tridentate tpy ligand compared to bidentate bpy ligands leads to a less active but potentially more stable H_2_-evolving catalyst. However, **Rh-tpy**, is much less efficient than the cobalt(III) tetraazamacrocyclic [Co^III^(CR14)Cl_2_]^+^ (**Co**) complex, ione of the most efficient H_2_-evolving catalysts in acidic water, since with this catalyst, TON_Cat_* of 1086 and 1822 were reached at 10 and 5 μM, respectively.

The electrochemical study in DMF reveals that the reduced state of the rhodium catalyst, identified as [Rh^I^(tpy)Cl] (**Rh^I^-tpy**) from its UV-visible signature, is stable for several hours under an inert atmosphere owing to the π-acceptor property of the terpyridine ligand that stabilizes the low oxidation states of the rhodium. A good stability of the low-valent form of the rhodium catalyst makes it less prone to degradation in the course of photocatalysis. The π-acceptor property of terpyridine also confers moderately negative reduction potential to the **Rh-tpy** catalyst (about −0.8 V vs. SCE), making its reduction by the reduced state of **Ru** effective (*E*_1/2_(Ru^II^/Ru^I^) = −1.50 V vs. SCE). A Stern–Volmer plot and transient absorption spectroscopy have shown that the first step of the photocatalytic process is a reductive quenching of the **Ru** excited state by ascorbate. The resulting **Ru^−^** species is then able to reduce the **Rh^III^-tpy** catalyst generating **Rh^I^-tpy,** which reacts with a proton on a sub-nanosecond time scale to form a **Rh^III^(H)-tpy** hydride, the key intermediate for H_2_ evolution. The search for new molecular catalysts for H_2_ production with simple and easily synthesized ligands is still ongoing, and the terpyridine ligand, with its particular electronic and coordination properties, is a good candidate for designing new catalysts to meet these requirements.

## Data Availability

Not applicable.

## Supplementary Materials

The following supporting information can be downloaded at: https://www.mdpi.com/article/10.3390/molecules27196614/s1, Materials and general experimental details. Additional data and experimental details for X-ray structure determination, electrochemistry, UV-Vis absorption spectroscopy, 1H NMR, mass spectrum, photocatalytic hydrogen production, photophysics, nanosecond transient absorption spectroscopy (Figures S1–S8 and Tables S1–S7). CCDC 2173038 contains the supplementary crystallographic data for this paper. These data can be obtained free of charge via www.ccdc.cam.ac.uk/data_request/cif or by emailing data_request@ccdc.cam.ac.uk, or by contacting The Cambridge Crystallographic Data Centre, 12 Union Road, Cambridge CB2 1EZ, U.K.; fax: +44-1223-336033.

## Sample Availability

Samples of the compounds [Rh^III^(tpy)Cl_3_] and [Rh^III^(tpy)(CH_3_CN)Cl_2_](CF_3_SO_3_) are available from the authors.
