# Supplementary material for: Electrochemical Properties of a Rhodium(III) Mono-Terpyridyl Complex and Use as a Catalyst for Light-Driven Hydrogen Evolution in Water
Source: Molecules. 2022 Oct 5;27(19):6614. doi: 10.3390/molecules27196614 (PMC9571878; doi:10.3390/molecules27196614)
Supplement: Supplementary file 1 [file molecules-27-06614-s001.zip › molecules-1917342-supplementary.pdf]

Supplementary Materials

Electrochemical Properties of a Rhodium(III) Mono-Terpyridyl Complex and use as a Catalyst for Light-Driven Hydrogen Evolution in Water

Fakourou Camara [1,2], Thomas Gavaggio [1], Baptiste Dautreppe [1], Jérôme Chauvin [1], Jacques Pécaut [2], Dmitry Aldakov [2], Marie-Noëlle Collomb [1,*] and Jérôme Fortage [1,*]

[1] DCM, CNRS, Université Grenoble Alpes, 38000 Grenoble, France
[2] SyMMES, IRIG, CEA, CNRS, Université Grenoble Alpes, 38000 Grenoble, France
* Correspondence: marie-noelle.collomb@univ-grenoble-alpes.fr (M.-N.C.); jerome.fortage@univ-grenoble-alpes.fr (J.F.)

Table of Contents

Materials and general experimental details……………………………………………………3

X-ray structure determination of [Rh(tpy)(CH_3CN)Cl_2](CF_3SO_3) (Rh-tpy)……………...4

Table S1. Crystallographic refinement data for [Rh(tpy)(CH_3CN)Cl_2](CF_3SO_3)……………..………4

Table S2. Bond lengths in Å for [Rh(tpy)(CH_3CN)Cl_2](CF_3SO_3)..……………....................………..5

Table S3. Bond Angles in ° for [Rh(tpy)(CH_3CN)Cl_2](CF_3SO_3)..……………..................…………..5

Table S4. Torsion Angles in ° for [Rh(tpy)(CH_3CN)Cl_2](CF_3SO_3) ……………...........................…..6

Electrochemistry and UV-Visible absorption spectroscopy………………………………..8

Table S5. Electrochemical potentials of **Rh-tpy**, **Rh2**, **Ru** and NaHA….………………......................8

General procedure for photocatalytic hydrogen generation……………………………….9

Table S6. Concentrations and corresponding masses for a 5 mL aqueous solution of H_2A and NaHA at different pH..……………......................………...………………......................…..…………………....9

Table S7. Photocatalytic activities towards hydrogen evolution of all systems studied, in terms of TON_{Cat}, TOF_{Cat} and n_{H2}..………………................……….….…………….................………..10

Stern-Volmer plot for the quenching of *Ru by Rh-tpy……………………………..……10

Nanosecond transient absorption spectroscopy…………………………………………...10

Figure S1: Transient absorption spectra recorded at different times after laser excitation (λ = 455 nm) of an aqueous solution containing **Ru** and NaHA/H_2A at pH 4.5……………………11

Figure S2: Transient absorption trace on the nanosecond time scale recorded at 510 nm after laser excitation at 455 nm of an aqueous solution containing **Ru** and NaHA/H_2A at pH 4.5………………...11

Figure S3: Transient absorption trace on the microsecond time scale recorded at 510 nm after laser excitation at 455 nm of an aqueous solution containing **Ru** and NaHA/H_2A at pH 4.5………………...12

Figure S4: Transient absorption trace on the nanosecond time scale recorded at 510 nm after laser excitation at 455 nm of a deaerated aqueous solution containing **Ru** (100 µM), NaHA/H_2A (0.1 M) and **Rh-tpy** (200 µM) at pH 4.5……………………………………………………………………12

Figure S5: Transient absorption trace on the microsecond time scale recorded at 510 nm after laser excitation at 455 nm of an aqueous solution containing **Ru**, NaHA/H_2A and **Rh-tpy** at pH 4.5………13

Estimation of the electron transfer rate between Ru^- and $HA^•$, and between Ru^- and Rh^{III}-tpy……………………………………………………………………………………14

Figure S6. ^{1}H NMR spectrum (400 MHz) in D_2O of [Rh(tpy)(CH_3CN)Cl_2](CF_3SO_3)………………..15

Figure S7. Positive ESI-MS spectrum of [Rh(tpy)(CH_3CN)Cl_2](CF_3SO_3)……………………………16

Figure S8. Experimental positive ESI-MS spectrum of [Rh(tpy)(CH_3CN)Cl_2](CF_3SO_3) and simulation of the main isotopic massif at *m/z* = 446.96 corresponding to [Rh(tpy)(CH_3CN)Cl_2]$^+$ ….………………...…………………………………………………………………………17

Scheme S1. Photosensitizers previously reported in three-component systems for light-driven H_2 production using Rh catalysts in aqueous or hydro-organic solutions………………………………..19

Table S8. Summary of the experimental conditions and photocatalytic performances of the various three-component systems (SD/PS/Cat) using Rh catalysts in hydro-organic solution………………20

Table S9. Summary of the experimental conditions and photocatalytic performances of various three-component systems (SD/PS/Cat) using Rh catalysts in pure aqueous solution………………………..22

References……………………………………………………………………………………23

Materials and general experimental details. Acetonitrile (CH_3CN, Fisher, HPLC grade), ethanol (EtOH, Fisher, HPLC grade), diethyl ether (Et_2O, 98%, VWR), N,N-dimethylformamide (DMF, Fisher, 99,9%, Carlo Erba), sodium trifluoromethanesulfonate ($NaCF_3SO_3$, 98 %, Aldrich), silver trifluoromethanesulfonate ($AgCF_3SO_3$, > 99 %, Aldrich), rhodium(III) chloride hydrate ($RhCl_3{\cdot}nH_2O$, 38%w Rh, Acros), 2,2';6',2''-terpyridine (tpy, 98%, Merck), dimethylsulfoxide-d^6 ($SO(CD_3)_2$, Euriso-top, 99.8%D), deuterium oxide-d_2 (D_2O, Euriso-top, 99.97%D), $[Ru(bpy)_3]Cl_2$ (**Ru**) (99%, Aldrich), L-ascorbic acid (H_2A, 99%, Fischer), sodium L-ascorbate (NaHA, 99%, Acros), sodium acetate (NaOAc, > 99%, Fischer), acetic acid (AcOH, > 98%, VWR) and reference gas (1% and 5% H_2 in N_2, Air Liquide) were purchased from commercial suppliers. All reagents and solvents were used as received. Purification of water (15.0 MΩ cm, 24°C) was performed with a milli-Q system (Purelab option, Elga). ^1H NMR spectra were recorded on a Bruker 400 MHz spectrometer. Chemical shifts for ^1H NMR spectra are referenced relative to residual protium in deuterated dimethylsulfoxide ($SO(CD_3)_2$, δ = 2.50 ppm) and deuterium oxide (D_2O, δ = 4.80 ppm). ^1H NMR and mass spectrometry were performed within the ICMG Chemistry Nanobio Platform, Grenoble. Electrospray ionisation mass spectrometry (ESI-MS) were performed on a Bruker Esquire 3000 Plus ion trap spectrometer equipped with an electrospray ion source (ESI). The samples were analysed in either positive or negative mode ionization mode as well as in MS^2 by direct perfusion in the ESI-MS interface. The analyses were carried out in DMF for $[Rh(tpy)Cl_3]$ and in a mixture of $CH_3CN/DMF/H_2O$ for $[Rh(tpy)(CH_3CN)Cl_2](CF_3SO_3)$ (**Rh-tpy**) at a complex concentration of approximately 1 µmol L^{-1}. The infrared (IR) spectra were recorded on a Perkin-Elmer Spectrum GX FT-IR spectrometer on powder samples in KBr pellets with a 2%w concentration.

X-ray structure determination of $[Rh(tpy)(CH_3CN)Cl_2](CF_3SO_3)$, denoted Rh-tpy. Single translucent light yellow prism-shaped crystals of $[Rh(tpy)(CH_3CN)Cl_2](CF_3SO_3)$ were obtained by recrystallization *via* a slow diffusion of diethyl ether into an acetonitrile solution of complex. A suitable crystal 0.16×0.15×0.07 mm^3 was selected and mounted on a suitable support on an Xcalibur, Sapphire3 diffractometer. The crystal was kept at a steady T = 150(1) K during data collection. The structure was solved with the ShelXS-97 (Sheldrick, 1990) structure solution program using the Intrinsic Phasing solution method and by using **Olex2** as the graphical interface [1]. The model was refined with version of XL n/a (Sheldrick, 2008) using Least Squares minimization.

Table S1. Crystallographic refinement data for the structure of $[Rh(tpy)(CH_3CN)Cl_2](CF_3SO_3)$

	$[Rh(tpy)(CH_3CN)Cl_2](CF_3SO_3)$
CDCD reference	2173038
Empirical formula	$C_{18}H_{14}Cl_2F_3N_4O_3RhS$
Formula weight	597.20
Colour, shape	Yellow, prism
Crystal size, mm	0.16 x 0.15 x 0.07
Crystal system	Triclinic
Space group	P 1
a, Å	8.6026(2)
b, Å	8.9644(2)
c, Å	14.5054(3)
α, deg.	106.371(2)
β, deg.	102.674(2)
γ, deg.	93.505(2)
V, Å^3	1038.04(4)
Z	2
T, K	150(1)
Radiation type	Mo K_α
Wavelength, Å	0.71073
ρ (calc), Mg/m^3	1.911
β mm^{-1}	1.238
θ range, deg.	2.39 to 30.51
Refl. collected/unique (R_{int})	25279/6335(0.0263)
Refl. observed with $I_0 > 2\sigma(I_0)$	5731
Data/restraints/parameters	6335/0/345
GooF	1.063
R1	0.0250
wR2	0.0566

Table S2. Bond lengths in Å for $[Rh(tpy)(CH_3CN)Cl_2](CF_3SO_3)$

Atom	Atom	Length
Rh1	Cl1	2.3530(5)
Rh1	Cl2	2.3067(4)
Rh1	N1	2.0258(14)
Rh1	N2	1.9386(14)
Rh1	N3	2.0417(14)
Rh1	N21	2.0220(14)
N1	C1	1.336(2)
N1	C5	1.367(2)
N2	C6	1.343(2)
N2	C10	1.341(2)
N3	C11	1.361(2)
N3	C15	1.342(2)
N21	C21	1.133(2)
C1	C2	1.383(3)
C2	C3	1.375(3)
C3	C4	1.387(3)
C4	C5	1.383(2)
C5	C6	1.476(2)

Atom	Atom	Length
C6	C7	1.388(2)
C7	C8	1.387(3)
C8	C9	1.380(3)
C9	C10	1.389(3)
C10	C11	1.475(3)
C11	C12	1.387(2)
C12	C13	1.388(3)
C13	C14	1.371(3)
C14	C15	1.389(3)
C21	C22	1.453(3)
S1	O1	1.4371(15)
S1	O2	1.4380(15)
S1	O3	1.4404(15)
S1	C1S	1.818(2)
F1	C1S	1.332(2)
F2	C1S	1.336(2)
F3	C1S	1.333(2)

Table S3. Bond Angles in ° for $[Rh(tpy)(CH_3CN)Cl_2](CF_3SO_3)$

Atom	Atom	Atom	Angle/°
Cl2	Rh1	Cl1	93.570(18)
N1	Rh1	Cl1	99.28(4)
N1	Rh1	Cl2	88.71(4)
N1	Rh1	N3	162.03(6)
N2	Rh1	Cl1	177.84(4)
N2	Rh1	Cl2	88.56(4)
N2	Rh1	N1	81.07(6)
N2	Rh1	N3	81.00(6)
N2	Rh1	N21	87.90(6)
N3	Rh1	Cl1	98.68(4)
N3	Rh1	Cl2	89.80(4)
N21	Rh1	Cl1	89.97(4)
N21	Rh1	Cl2	176.01(4)
N21	Rh1	N1	88.90(5)
N21	Rh1	N3	91.48(5)
C1	N1	Rh1	127.02(12)
C1	N1	C5	120.10(15)
C5	N1	Rh1	112.88(11)
C6	N2	Rh1	117.63(11)
C10	N2	Rh1	117.91(12)
C10	N2	C6	124.44(15)
C11	N3	Rh1	112.48(11)
C15	N3	Rh1	127.32(13)
C15	N3	C11	120.19(15)
C21	N21	Rh1	170.48(14)
N1	C1	C2	121.27(18)
C3	C2	C1	119.06(18)
C2	C3	C4	120.05(17)
C5	C4	C3	118.67(18)
N1	C5	C4	120.60(16)
N1	C5	C6	114.54(14)

Atom	Atom	Atom	Angle/°
C4	C5	C6	124.83(16)
N2	C6	C5	113.34(14)
N2	C6	C7	118.71(16)
C7	C6	C5	127.93(16)
C8	C7	C6	118.16(18)
C9	C8	C7	121.63(18)
C8	C9	C10	118.47(18)
N2	C10	C9	118.52(17)
N2	C10	C11	113.22(15)
C9	C10	C11	128.24(16)
N3	C11	C10	115.32(14)
N3	C11	C12	120.55(18)
C12	C11	C10	124.11(17)
C11	C12	C13	119.16(19)
C14	C13	C12	119.59(17)
C13	C14	C15	119.51(19)
N3	C15	C14	120.96(19)
N21	C21	C22	178.53(18)
O1	S1	O2	115.53(9)
O1	S1	O3	115.08(10)
O1	S1	C1S	103.09(10)
O2	S1	O3	114.62(10)
O2	S1	C1S	103.01(9)
O3	S1	C1S	102.97(9)
F1	C1S	S1	112.08(14)
F1	C1S	F2	107.29(17)
F1	C1S	F3	107.07(16)
F2	C1S	S1	111.61(13)
F3	C1S	S1	111.49(14)
F3	C1S	F2	107.01(16)

Table S4. Torsion Angles in ° for [Rh(tpy)(CH_3CN)Cl_2](CF_3SO_3)

Atom	Atom	Atom	Atom	Angle/°
Rh1	N1	C1	C2	-177.09(14)
Rh1	N1	C5	C4	174.54(13)
Rh1	N1	C5	C6	-7.48(17)
Rh1	N2	C6	C5	1.89(18)
Rh1	N2	C6	C7	-179.29(12)
Rh1	N2	C10	C9	-178.44(12)
Rh1	N2	C10	C11	3.01(18)
Rh1	N3	C11	C10	-0.30(17)
Rh1	N3	C11	C12	-179.15(13)
Rh1	N3	C15	C14	179.05(12)
Rh1	N21	C21	C22	116(7)
Cl1	Rh1	N1	C1	8.79(15)
Cl1	Rh1	N1	C5	-171.21(10)
Cl1	Rh1	N2	C6	94.7(12)
Cl1	Rh1	N2	C10	-84.2(12)
Cl1	Rh1	N3	C11	179.29(10)
Cl1	Rh1	N3	C15	-1.44(14)
Cl1	Rh1	N21	C21	169.0(8)
Cl2	Rh1	N1	C1	-84.61(15)
Cl2	Rh1	N1	C5	95.38(11)
Cl2	Rh1	N2	C6	-93.58(12)
Cl2	Rh1	N2	C10	87.50(12)
Cl2	Rh1	N3	C11	-87.13(11)
Cl2	Rh1	N3	C15	92.14(14)
Cl2	Rh1	N21	C21	16.4(13)
N1	Rh1	N2	C6	-4.66(12)
N1	Rh1	N2	C10	176.42(12)
N1	Rh1	N3	C11	-1.9(2)
N1	Rh1	N3	C15	177.34(16)
N1	Rh1	N21	C21	69.8(8)
N1	C1	C2	C3	1.6(3)
N1	C5	C6	N2	3.9(2)
N1	C5	C6	C7	-174.78(16)
N2	Rh1	N1	C1	-173.36(15)
N2	Rh1	N1	C5	6.63(11)
N2	Rh1	N3	C11	1.45(11)
N2	Rh1	N3	C15	-179.28(15)
N2	Rh1	N21	C21	-11.3(8)
N2	C6	C7	C8	-1.5(2)
N2	C10	C11	N3	-1.7(2)
N2	C10	C11	C12	177.13(16)
N3	Rh1	N1	C1	-169.98(16)
N3	Rh1	N1	C5	10.0(2)
N3	Rh1	N2	C6	176.39(12)
N3	Rh1	N2	C10	-2.53(12)
N3	Rh1	N21	C21	-92.3(8)
N3	C11	C12	C13	-0.1(3)
N21	Rh1	N1	C1	98.59(15)
N21	Rh1	N1	C5	-81.42(11)
N21	Rh1	N2	C6	84.56(12)

N21	Rh1	N2	C10	-94.36(12)
N21	Rh1	N3	C11	89.09(11)
N21	Rh1	N3	C15	-91.64(14)
C1	N1	C5	C4	-5.5(2)
C1	N1	C5	C6	172.51(15)
C1	C2	C3	C4	-3.6(3)
C2	C3	C4	C5	1.1(3)
C3	C4	C5	N1	3.4(3)
C3	C4	C5	C6	-174.35(16)
C4	C5	C6	N2	-178.21(16)
C4	C5	C6	C7	3.1(3)
C5	N1	C1	C2	2.9(3)
C5	C6	C7	C8	177.12(16)
C6	N2	C10	C9	2.7(2)
C6	N2	C10	C11	-175.83(14)
C6	C7	C8	C9	1.2(3)
C7	C8	C9	C10	1.1(3)
C8	C9	C10	N2	-2.9(3)
C8	C9	C10	C11	175.36(17)
C9	C10	C11	N3	179.94(16)
C9	C10	C11	C12	-1.3(3)
C10	N2	C6	C5	-179.27(14)
C10	N2	C6	C7	-0.5(2)
C10	C11	C12	C13	-178.82(16)
C11	N3	C15	C14	-1.7(2)
C11	C12	C13	C14	-1.2(3)
C12	C13	C14	C15	1.0(3)
C13	C14	C15	N3	0.5(3)
C15	N3	C11	C10	-179.63(14)
C15	N3	C11	C12	1.5(2)
O1	S1	C1S	F1	61.60(16)
O1	S1	C1S	F2	-178.03(14)
O1	S1	C1S	F3	-58.41(16)
O2	S1	C1S	F1	-177.88(14)
O2	S1	C1S	F2	-57.51(16)
O2	S1	C1S	F3	62.11(16)
O3	S1	C1S	F1	-58.41(17)
O3	S1	C1S	F2	61.96(17)
O3	S1	C1S	F3	-178.42(15)

Electrochemistry and UV-Visible absorption spectroscopy. The electrochemical measurements were run under an argon atmosphere into a dry-glove box at room temperature. Cyclic voltammetry and electrolysis experiments were performed using a Solartron Analytical Instrument potentiostat (Modulab XM MTS) with XM-studio as software. A standard three-electrode electrochemical cell was used and electrochemical experiments were carried out in DMF (7 mL) with 0.1 M $[Bu_4N]ClO_4$ as electrolyte and 0.5 mM of **Rh-tpy**. The working electrode, polished with 2 µm diamond paste (MecaprexPresi), was a carbon vitreous disk (3 mm in diameter) for cyclic voltammetry ($E\text{p}_a$, anodic peak potential; $E\text{p}_c$, cathodic peak potential; $E_{1/2} = (E\text{p}_a + E\text{p}_c)/2$; $\Delta E\text{p} = E\text{p}_a - E\text{p}_c$). Exhaustive electrolyses were carried out on a reticulated vitreous carbon electrode 60 PPI (the electrosynthesis Co. Inc.) three-dimensional meshes (1.8 cm x 0.7 cm x 0.4 cm). The auxiliary electrode was a Pt wire in DMF + 0.1 M $[Bu_4N]ClO_4$. Potentials were referred to an $Ag/AgNO_3$ 0.01 M reference electrode in CH_3CN + 0.1 M $[Bu_4N]ClO_4$. The potential of our reference electrode Ag^+ (0.01 M $AgNO_3$, 0.1 M $[Bu_4N]ClO_4$, CH_3CN)/Ag was carefully checked at the end of each experiment by adding ferrocene. The potential of the ferrocenium/ferrocene ($Fc^{+/0}$) couple (reversible wave) appears at $E_{1/2}$ = +100 mV. Potentials referred to our $Ag/AgNO_3$ can be thus converted to the $Fc^{+/0}$ couple by subtracting 100 mV and converted to Saturated Calomel Electrode (SCE) by adding 298 mV. According to Pavlishchuk et al. [2], the potential of $Fc^{+/0}$ couple is +87 mV *versus* a reference electrode Ag^+ (0.01 M $AgNO_3$, 0.1 M $[Et_4N]ClO_4$, CH_3CN)/Ag. The UV-Vis spectra were recorded with a MCS 501 UV-NIR (Carl Zeiss) spectrophotometer equipped with an automatic shutter. The light sources are halogen (CLH 500 20 W) and deuterium lamps (CLD 500) with optic fibers (041.002–UV SN 012105) in order to perform the spectroelectrochemical experiments into the glove box with a 1 mm path-length probe.

Table S5. Electrochemical potentials of **Rh-tpy**, **Rh2**, **Ru** and NaHA. Potentials are referred *versus* Ag/0.01 M $AgNO_3$ in acetonitrile and *versus* Saturated Calomel Electrode (SCE) in water.

Compound	Solvent	Redox potentials/V (ΔE_p/mV) *vs* Ag/0.01 M $AgNO_3$		
$[Rh^{III}(tpy)(CH_3CN)Cl_2](CF_3SO_3)$<br>(**Rh-tpy**)[This work]	DMF, 0.1M<br>$[Bu_4N]ClO_4$	$E\text{p}_c$= -1.13 V<br>$E\text{p}_a$= -0.72 V<br>(Rh^{III}/Rh^I)	$E_{1/2}$ = -1.95 V<br>(130)<br>($tpy/tpy^{\bullet -}$)	
$[Rh^{III}(dmbpy)_2Cl_2](BF_4)$<br>(**Rh2**)[3]	CH_3CN,<br>0.1M<br>$[Bu_4N]ClO_4$	$E\text{p}_c$= -1.28 V<br>$E\text{p}_a$= -0.72 V<br>(Rh^{III}/Rh^I)	$E_{1/2}$ = -1.77 V (70)<br>($bpy/bpy^{\bullet -}$)	$E_{1/2}$ = -2.01 V (60)<br>($bpy/bpy^{\bullet -}$)
$[Ru(bpy)_3]Cl_2$ (**Ru**)[4]	CH_3CN,<br>0.1M<br>$[Bu_4N]ClO_4$	$E_{1/2}$ = +0.98 V<br>(80)<br>(Ru^{III}/Ru^{II})	$E_{1/2}$ = -1.64 V (60)<br>(Ru^{II}/Ru^-)	$E_{1/2}$ = -1.83 (60) and<br>-2.08 V (70)<br>($bpy/bpy^{\bullet -}$)
Compound	Solvent	Redox potentials/V (ΔE_p/mV) *vs* SCE		
$[Rh^{III}(dmbpy)_2Cl_2](Cl)$<br>(**Rh2**)[3]	H_2O<br>(pH 8)	$E\text{p}_c$= -0.79 V<br>$E\text{p}_a$= -0.25 V<br>(Rh^{III}/Rh^I)		
$[Ru(bpy)_3]Cl_2$ (**Ru**)[5, 6]	H_2O<br>(pH 7)	$E_{1/2}$ = +1.03 V<br>(Ru^{III}/Ru^{II})	$E_{1/2}$ = -1.50 V<br>(Ru^{II}/Ru^-)	
*$[Ru(bpy)_3]Cl_2$ (***Ru**)[5, 6], [a]	H_2O<br>(pH 7)	-1.07 V<br>($Ru^{III}/{}^*Ru^{II}$)	+0.60 V<br>($^*Ru^{II}/Ru^-$)	
HA^-[7]	H_2O<br>(pH 3.58)	$E\text{p}_a$ = +0.11 V		

[a] Oxidation and reduction potentials of the excited state of **Ru** (denoted ***Ru**) are estimated by using the formula: $E_{1/2}(Ru^{III}/{}^*Ru^{II}) = E_{1/2}(Ru^{III}/Ru^{II}) - E_{0,0}$ and $E_{1/2}({}^*Ru^{II}/Ru^-) = E_{1/2}(Ru^{II}/Ru^-) + E_{0,0}$. $E_{0,0}$ is the energy level of the relaxed triplet excited state (^{3}MLCT) of **Ru** in water, which is equal to 2.1 eV.

General procedure for photocatalytic hydrogen generation. A 5 mL graduated flask was charged with the $[Ru^{II}(bpy)_3](Cl)_2$ (**Ru**) photosensitizer weighted as a solid (1.98 mg), in order to obtain a **Ru** concentration of 530 µM. Then 14, 71 and 143 µL of a rhodium catalyst solution ($[Rh^{III}(tpy)(MeCN)Cl_2](CF_3SO_3)$ (**Rh-tpy**) or $[Rh^{III}(dmbpy)_2Cl_2]Cl$ (**Rh2**) at 0.35 mM) in Milli-Q water or an aqueous 1 M acetate buffer (at pH 4.0, 4.5 or 4.8) were added into the 5 mL flask containing Ru in order to obtain a rhodium concentration of 1, 5 and 10 µM, respectively. After completing the volume to 5 mL with Milli-Q or 1 M acetate buffer, the **Ru** concentration (530 µM) was checked by UV-Vis using a cuvette of 1 mm optical pathlength (absorbance of 0.77 at 452 nm, ε = 14 600 M^{-1} cm^{-1}). The solution was then transferred in a homemade glass tube with a diameter of 2 cm fused with a round bottom flask (head space volume of 165 mL for experiments with **Rh-tpy** and 193 mL for experiments with **Rh2**) containing a magnetic stirrer (volume < 0.1 mL),sodium ascorbate (NaHA) and ascorbic acid (H_2A), as solids and with the appropriate and respective concentrations in function of pH (4.0, 4.5 or 4.8) and the total NaHA/H_2A concentration used (*i.e.* 1.1 M for aqueous solutions with only Milli-Q water or 0.1 M for aqueous solutions of 1 M acetate buffer, see Table S6). The tube was sealed with a rubber septum, protected from light with aluminum foil before irradiation experiments, and the solution was degassed for 45 min by argon bubbling.

Aqueous solutions of 1 M acetate buffer at different pHs (4.0, 4.5 or 4.8) were prepared by mixing AcOH (0.183 mL, 0.64 M) and NaOAc (0.148 g, 0.36 M) in Milli-Q water. Then the pH was adjusted to desired value by adding suitable amounts of NaOH or HCl.

For photocatalysis, continuous irradiation was performed at 298 K under stirring with a xenon lamp (150 W, Hamamatsu L8253, type LC8-03) equipped with a 400 – 700 nm large band filter which was placed 4 cm from the sample (P = 100 mW.cm^{-2} measured with a calibrated Avantes spectrometer AvaSpec-ULS2048CL-EVORS-UA, with a slit of 20 µM, equipped with a fiber cable FC-UVIR400-1 and a Cosine corrector CC-UV/VIS/NIR-8MM). The amount of hydrogen evolved was quantified from an analysis of the gas mixture in the headspace of the glass tube (sampling of 100 µL of gas) by gas chromatography (Perkin Elmer Autosystem XL Gas Chromatograph equipped with a 5 Å molecular sieve column (oven temperature = 303 K) and a thermal conductivity detector (TCD)), which uses argon as a carrier gas. Prior to each experiment, GC/TCD calibration was carried out by using two samples of the reference gas (1.045 % and 4.967 % H_2 in N_2).

The turnover number (TON) and turnover frequency (TOF) for H_2 production were determined as previously described [3, 8].

Table S6. Concentrations and corresponding masses for a 5 mL aqueous solution of ascorbic acid (H_2A) and sodium ascorbate (NaHA) at different pH with total NaHA/H_2A concentrations of 1.1[a] and 0.1 M[b] considering a pK_A of 4.1 for the couple NaHA/H_2A.

pH	NaHA	H_2A
4.0[a]	0.487 M (482 mg)	0.613 M (540 mg)
4.0[b]	44.3 mM (44 mg)	55.7 mM (49 mg)
4.5[b]	71.5 mM (71 mg)	28.5 mM (25 mg)
4.8[b]	83.4 mM (83 mg)	16.6 mM (15 mg)

Table S7. Photocatalytic activities towards hydrogen evolution of all systems studied in this work, in terms of TON_{Cat}, TOF_{Cat},n_{H2} and V_{H2}Experiments were carried out at 25° C in an deaerated aqueous solution (5 mL) buffered with 1.1 M NaHA/H_2A[a] or with 1 M sodium acetate and 0.1 M NaHA/H_2A,[b] containing the $[Ru^{II}(bpy)_3](Cl)_2$ (**Ru**, 530 μM) photosensitizer (PS) and a catalyst (Cat), $[Rh^{III}(tpy)(CH_3CN)Cl_2](CF_3SO_3)$ (**Rh-tpy**), $[Rh^{III}(dmbpy)_2Cl_2]Cl$ (**Rh2**), or $[Co^{III}(CR14)Cl_2]Cl$ (**Co**) previously reported {Gueret, 2018 #46} at various concentrations under visible-light (λ = 400 -700 nm) irradiation.

PS [μM]	Cat [μM]	pH	PS/Cat ratio	TON_{Cat}[c] (TON_{Cat}*)[d]	Initial TOF_{Cat}[c]	n_{H2}[c] (n_{H2}*)[d] μmol	v_{H2}[c](v_{H2}*)[d] mL	Irrad. Time[e]
**Ru** (530)	**-**	4.5[b]	-	-	-	6.14	0.15	21.0 h
**Ru** (530)	**Rh-tpy** (10)	4.0[a]	53/1	220	95	11.0	0.27	21.7 h
**Ru** (530)	**Rh-tpy** (10)	4.0[b]	53/1	379	38	18.9	0.46	22.2 h
**Ru** (530)	**Rh-tpy** (10)	4.5[b]	53/1	423 (300)	21	21.1 (14.96)	0.52 (0.37)	21.5 h
**Ru** (530)	**Rh-tpy** (10) with Hg	4.5[b]	53/1	433 (309)	39	21.6 (15.46)	0.53 (0.38)	23.0 h
**Ru** (530)	**Rh-tpy** (10)	4.8[b]	53/1	333	26	16.7	0.27	22.7 h
**Ru** (530)	**Rh-tpy** (1)	4.5[b]	530/1	2242 (1012)	2000	11.2 (5.06)	0.27 (0.12)	22.3 h
**Ru** (530)	**Rh-tpy** (5)	4.5[b]	106/1	804 (558)	293	20.1 (13.96)	0.49 (0.34)	25.0 h
**Ru** (530)	**Rh2** (1)	4.5[b]	530/1	2010 (772)	6664	10.0 (3.86)	0.24 (0.09)	22.0 h
**Ru** (530)	**Rh2** (5)	4.5[b]	106/1	1023 (778)	1492	25.6 (19.46)	0.62 (0.48)	24.0 h
**Ru** (530)	**Rh2** (10)	4.5[b]	53/1	1261 (1139)	639	63.1 (56.96)	1.55 (1.40)	21.7 h
**Ru** (500)	**-**	4.5	-	-	-		0.13	21 h
**Ru** (500)	**Co** (5)	4.5	100/1	2034 (1822)	2250	51.0 (45.7)	1.25 (1.12)	22 h
**Ru** (500)	**Co** (10)	4.5	50/1	1192 (1086)	900	59.6 (54.3)	1.46 (1.33)	22 h
**Ru** (500)	**Co** (50)	4.5	10/1	912 (890)	240	228.2 (222.9)	5.59 (5.46)	22 h

[c]TON_{Cat} and TOF_{Cat} are respectively the maximum turnover number and the initial turnover frequencies (TOF_{Cat} = TON_{Cat} h^{-1}) per catalyst exhibited by the system, n_{H2} and V_{H2} the total mole number and volume of H_2 produced by the system until gas evolution stopped. [d]TON_{Cat}*,n_{H2}* and V_{H2}* are the corrected values of TON_{Cat}, n_{H2} and v_{H2}, respectively, obtained by subtracting the production of H_2 stemming from **Ru** without catalyst. [e]Irrad. time: time of irradiation after which H_2 production stopped.

Stern-Volmer plot for the quenching of *Ru by Rh-tpy. The Stern-Volmer experiments were carried out with several deaerated aqueous solutions (1 M acetate buffer at pH 4.5) containing **Ru** (10 μM) in presence of **Rh-tpy** at different concentrations (0; 0.06; 0.08; 0.1; 0.15; 0.2; 0.3 mM); the latter solutions were prepared in a glove box under argon atmosphere within quartz cells (cell width = 1 x 1 cm^2). The emission decays (τ) of the **Ru** excited state (***Ru**) were recorded after excitation at 409 nm with a Picosecond Pulsed Diode Laser (PDL 800D PicoQuant, Diode Laser: LDH-P-C-405) and using a time correlated single photon counting detection (picoHarp 300). The lifetimes of ***Ru** (τ) in presence of **Rh-tpy**, estimated from the fitting of the decay of ***Ru** luminescence at 610 nm, were used to calculate the τ_0/τ value to trace the Stern Volmer plot; τ_0 is the lifetime of ***Ru** alone (τ_o = 620 ns). The quenching of ***Ru** by **Rh-tpy** was estimated to be 6.87 x 10^8 M^{-1} s^{-1} (Figure 9).

Nanosecond transient absorption spectroscopy. The photocatalytic mechanism was studied by nanosecond laser flash photolysis experiments which were performed by exciting at 455 nm a deaerated aqueous acetate (1 M) buffer solutions at pH 4.5 containing **Ru** (100 µM) and NaHA/H_2A (0.1 M), in presence or absence of **Rh-tpy** (200 µM). Samples were prepared in a glove box under an argon atmosphere within a quartz cell (cell width = 10 × 10 mm^2). Transient absorption spectra were acquired using a LP 920K system from Edinburgh Instruments. Excitation at 455 nm was carried out from the third-harmonic of a Brilliant-Quantel Nd:YAG Laser at 6 Hz, equipped with an OPO (Rainbow). A Xe900 pulsed Xenon Lamp is used as probe source. The photons were dispersed using a monochromator, transcripted by a R928 (Hamamatsu) photomultiplicator and recorded on a TDS3012C (Tektronix) oscilloscope. Single wavelengths as well as global analyses of the transient absorption data were performed using Igor Pro wavemetrics software.

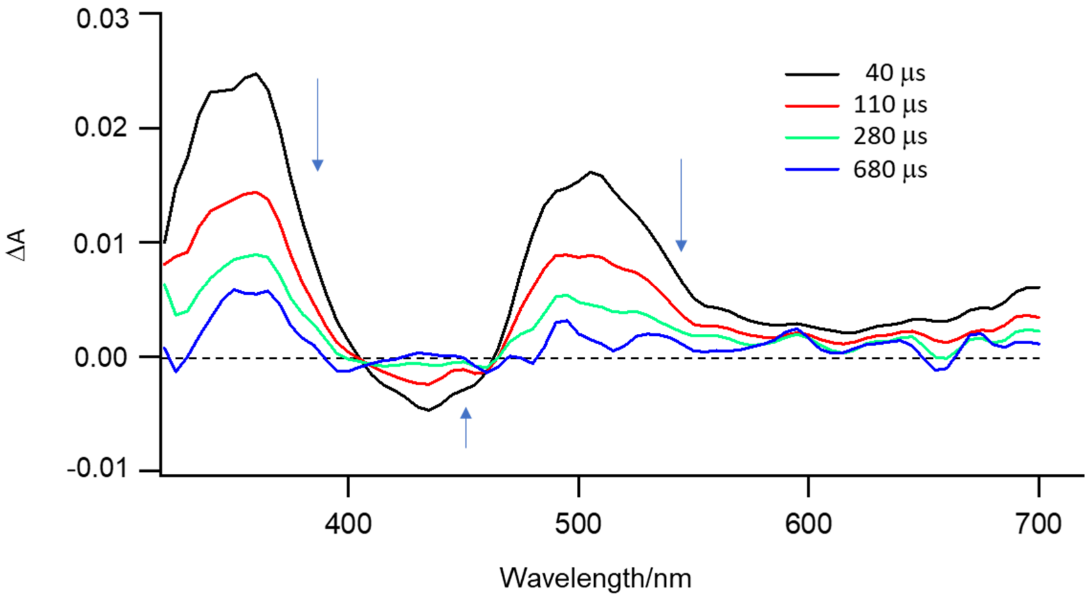

Figure S1: Transient absorption spectra recorded at different times after laser excitation (λ = 455 nm) of a deaerated aqueous solution of acetate buffer (1M) containing **Ru** (100 µM) and NaHA/H_2A (0.1 M) at pH 4.5 (pathlength = 1 cm). The signal growth at 510 nm occurs within 1.5 µs after laser excitation (see Figure S2).

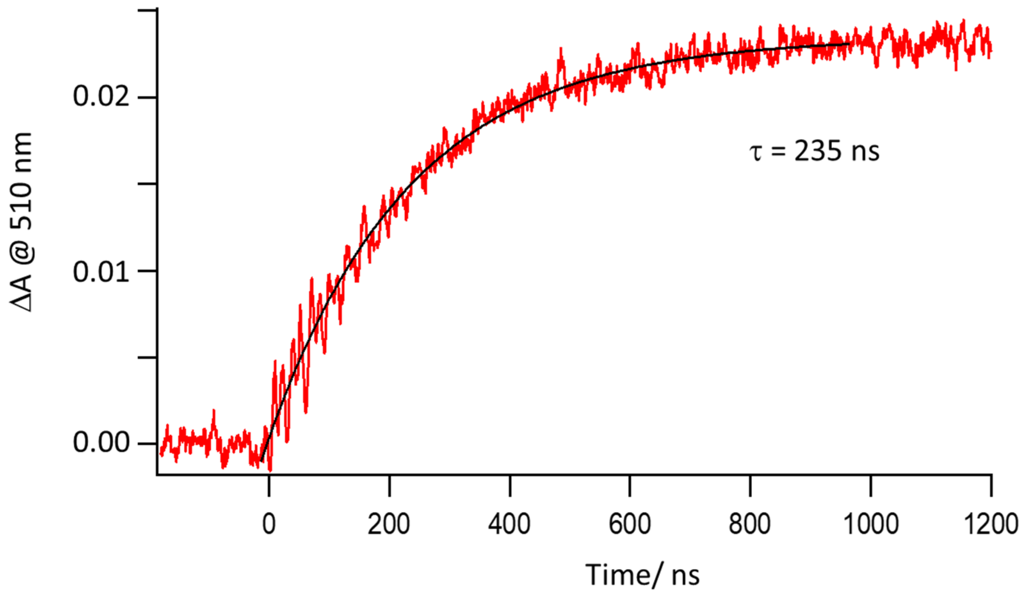

Figure S2: Transient absorption trace on the nanosecond time scale recorded at 510 nm after laser excitation at 455 nm of a deaerated aqueous solution of acetate buffer (1M) containing **Ru** (100 µM) and NaHA/H_2A (0.1 M) at pH 4.5. the growth of ΔA is fitted by a monoexponential function leading to a time constant of 235 ns (*i.e.* k = 4.25 x 10^6 s^{-1}). After 1.5 µs, the signal starts to decay.

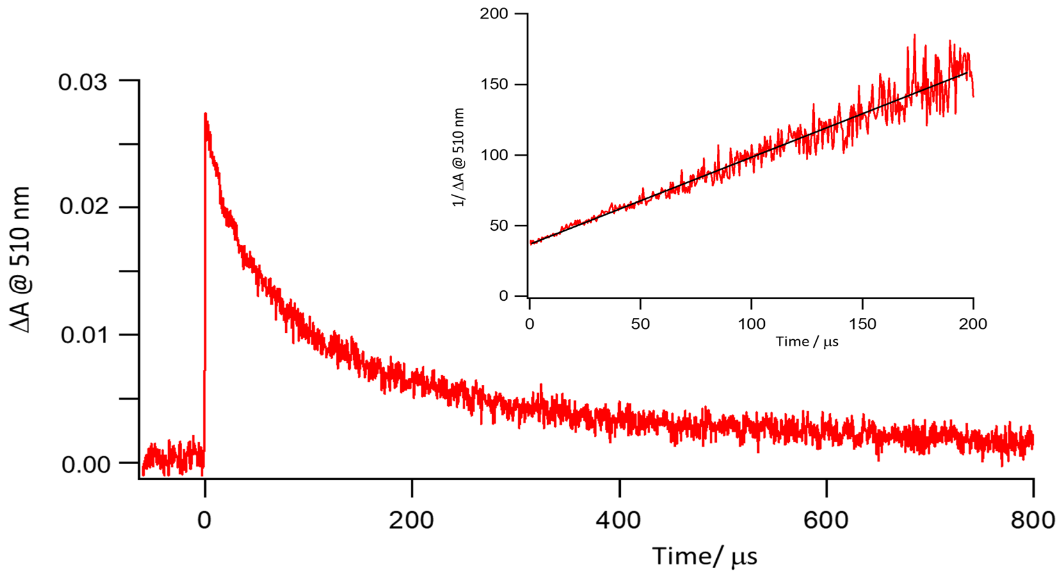

Figure S3: Transient absorption trace on the microsecond time scale recorded at 510 nm after laser excitation at 455 nm of a deaerated aqueous solution of acetate buffer (1M) containing **Ru** (100 µM) and NaHA/H_2A (0.1 M) at pH 4.5. Inset: second order kinetics analysis of the decay. The slope gives k_{BET} / (Δε λ) = 0.62 × 10^6 s^{-1}. Assuming Δε to 1.2 × 10^4 M^{-1} cm^{-1},[9] the kinetics rate constant for the back electron transfer process is estimated to 7.4 × 10^9 $M^{-1}s^{-1}$.

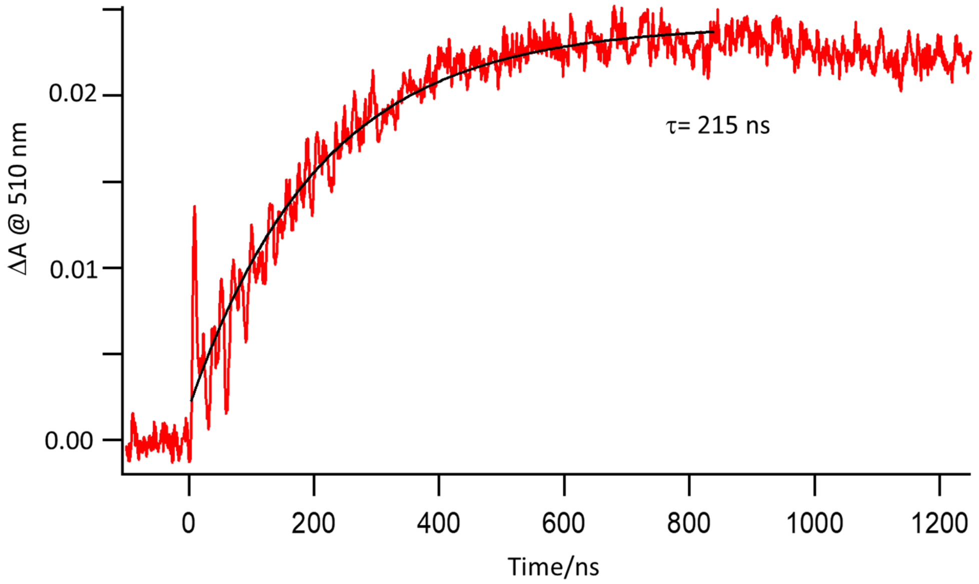

Figure S4: Transient absorption trace on the nanosecond time scale recorded at 510 nm after laser excitation at 455 nm of a deaerated aqueous solution of acetate buffer (1M) containing **Ru** (100 µM), NaHA/H_2A (0.1 M) and **Rh-tpy** (200 µM) at pH 4.5. the growth of ΔA is fitted by a monoexponential function leading to a time constant of 215 ns. After 1.5 µs the signal starts to decay.

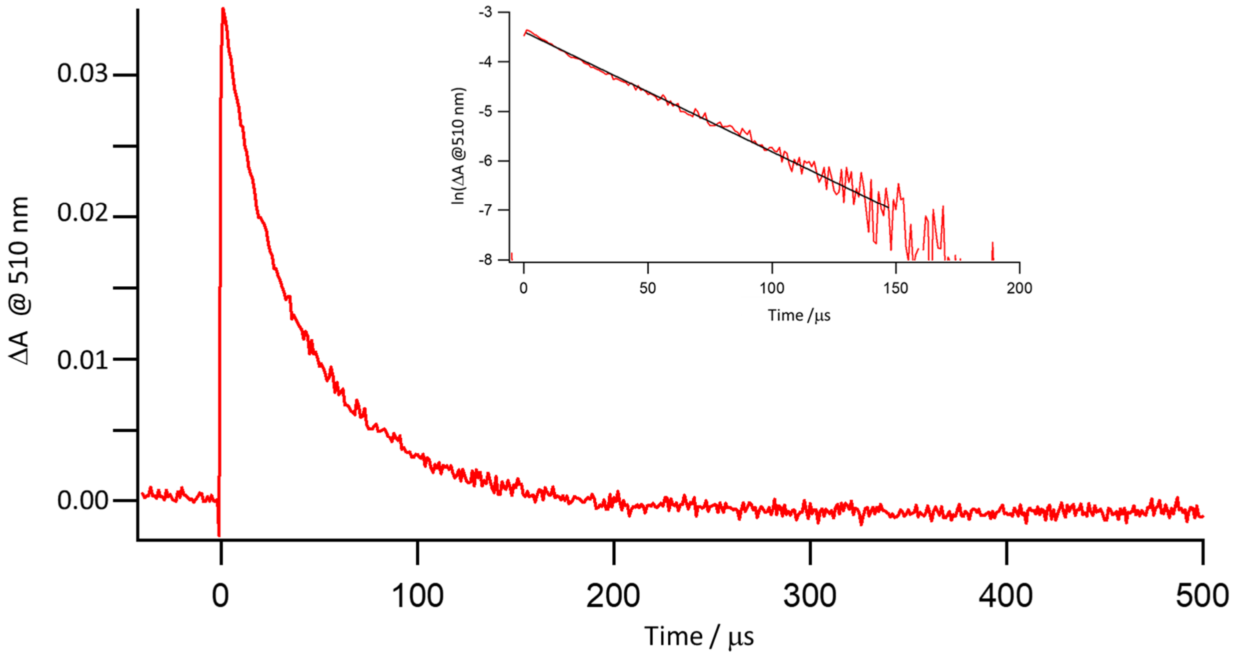

Figure S5: Transient absorption trace on the microsecond time scale recorded at 510 nm after laser excitation at 455 nm of a deaerated aqueous solution of acetate buffer (1M) containing **Ru** (100 µM), NaHA/H_2A (0.1 M) and **Rh-tpy** (200 µM) at pH 4.5. Inset: pseudo first order kinetics analysis of the decay. The slope gives a rate constant of 2.4 10^4 s^{-1}.

Estimation of the electron transfer rate between Ru⁻ and HA•, and between Ru⁻ and Rh^{III}-tpy. After the electron transfer from the sacrificial electron donor HA^- to the **Ru** excited state (***Ru^{II}**) to generate the reduced state **Ru⁻** and HA•, two processes of electron transfer are in competition (See Scheme 4): the back electron transfer (BET) between **Ru⁻** and HA• and the reduction of **Rh^{III}-tpy** by **Ru⁻** (ET). The rates (v) of these two redox reactions can be expressed as:

$$v_{BET} = k_{BET}[\mathbf{Ru^-}][HA^\bullet] \qquad (1)$$

$$v_{ET} = k_{ET}[\mathbf{Ru^-}][\mathbf{Rh^{III}\text{-}tpy}] \qquad (2)$$

Where v_{BET} and v_{ET} are the rates of these two redox reactions (M s^{-1}), k_{BET} and k_{ET} are the rate constants of these two redox reactions (respectively 7.4 x 10^9 and 1.2 x 10^8 M^{-1} s^{-1}), and [**Ru⁻**], [HA•] and [**Rh^{III}-tpy**] are the concentrations of the respective species. [**Rh^{III}-tpy**] is equal to 200 µM.

Considering that the quantum yield of **Ru⁻** formation *via* the reductive quenching of ***Ru^{II}** by HA^- is 25 % [10], the concentration [**Ru⁻**] can be estimated from the concentration [***Ru^{II}**], which can be calculated from the variation of the absorption (ΔA_{450} = -0.01) recorded at 450 nm just after the laser excitation (around 10 ns) in the transient absorption spectra of the solution **Ru** (100 µM)/H_2A/HA^- (0.1 M) (of note, the ΔA value is similar for the solution **Ru** (100 µM)/**Rh-tpy** (200 µM)/H_2A/HA^- (0.1 M)), and the variation of the extinction coefficient at 450 nm of the solution ($\Delta\varepsilon_{450} = \varepsilon_{450}^{Ru*} - \varepsilon_{450}^{Ru}$ = -11 000 M^{-1} cm^{-1}) [11]. So, following the Beer-Lambert law, the concentration [***Ru^{II}**] could be expressed as:

$$[\mathbf{*Ru^{II}}] = \Delta A_{450} / (\Delta\varepsilon_{450} \times l) \qquad (3)$$

Where l is the optical pathlength, l = 1 cm. Consequently, [***Ru^{II}**] can be estimated at 9.1 x 10^{-7} M and [**Ru⁻**] at 2.27 x 10^{-7} M.

In addition, just after the first electron transfer between HA^- and ***Ru^{II}**, we can consider that the concentration of HA• is equal to **Ru⁻** (*i.e.* [**Ru⁻**] = [HA•]).

Considering all these values, the values of v_{BET} and v_{ET} are estimated at 3.81 x 10^{-4} and 5.45 x 10^{-3} M s^{-1}, which corresponds to a v_{ET}/v_{BET} ratio of 14.3.

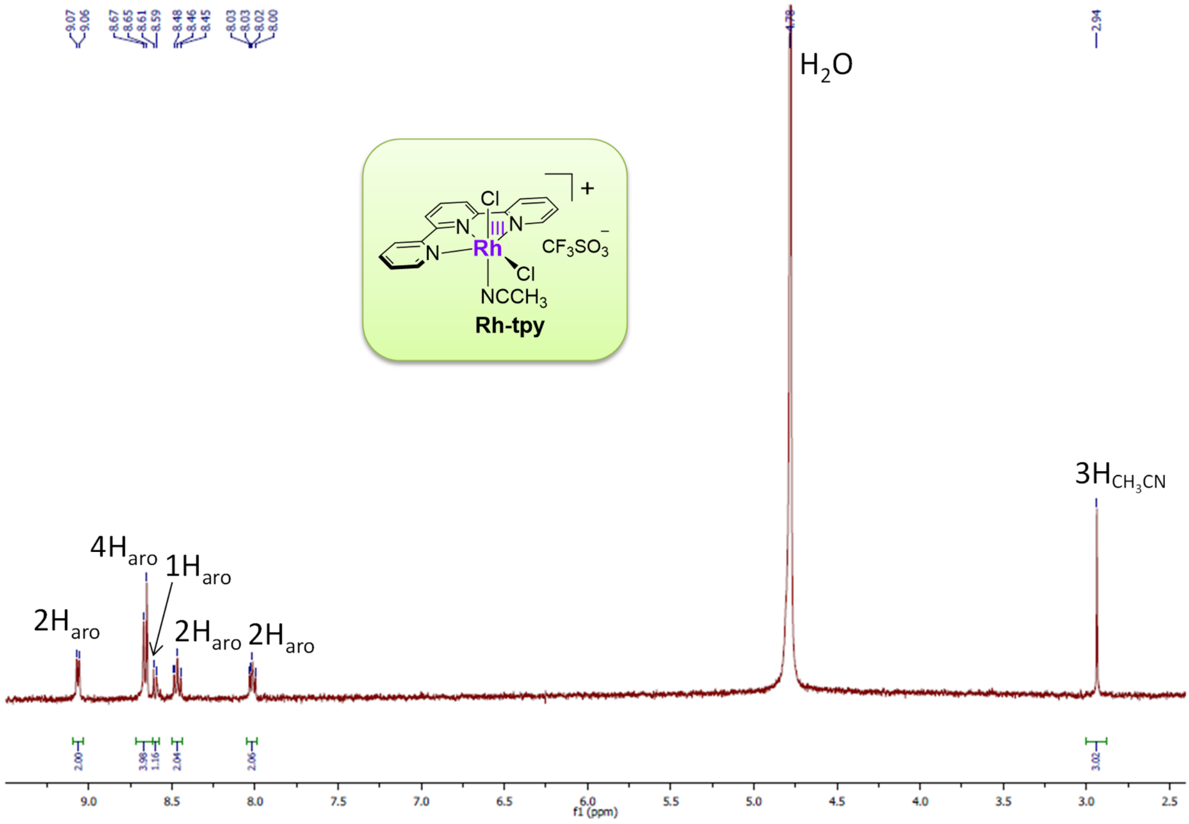

Figure S6. ^{1}H NMR spectrum (400 MHz) in D_2O of $[Rh(tpy)(CH_3CN)Cl_2](CF_3SO_3)$.

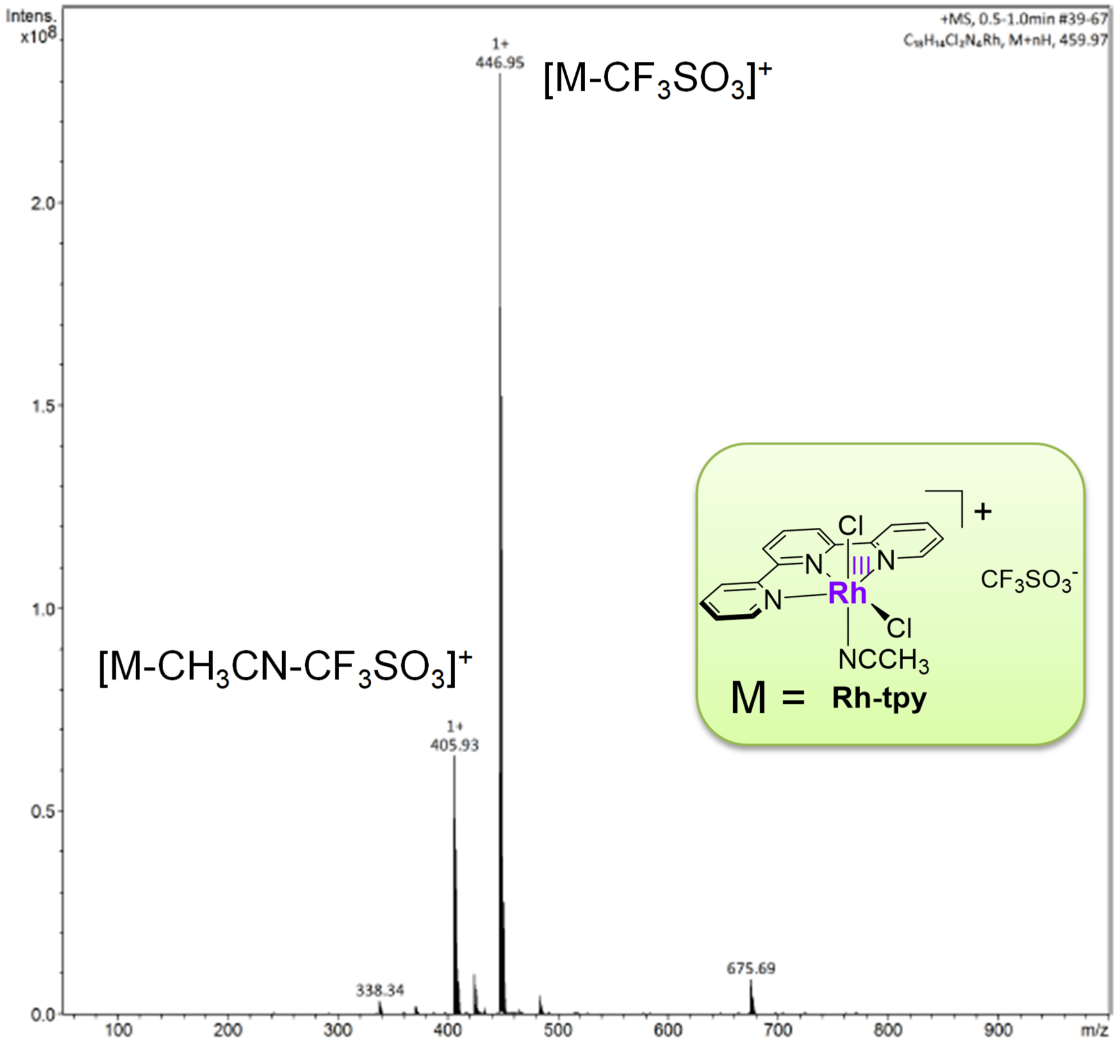

Figure S7. Positive ESI-MS spectrum of [Rh(tpy)(CH_3CN)Cl_2](CF_3SO_3) in a mixture of CH_3CN/DMF/H_2O.

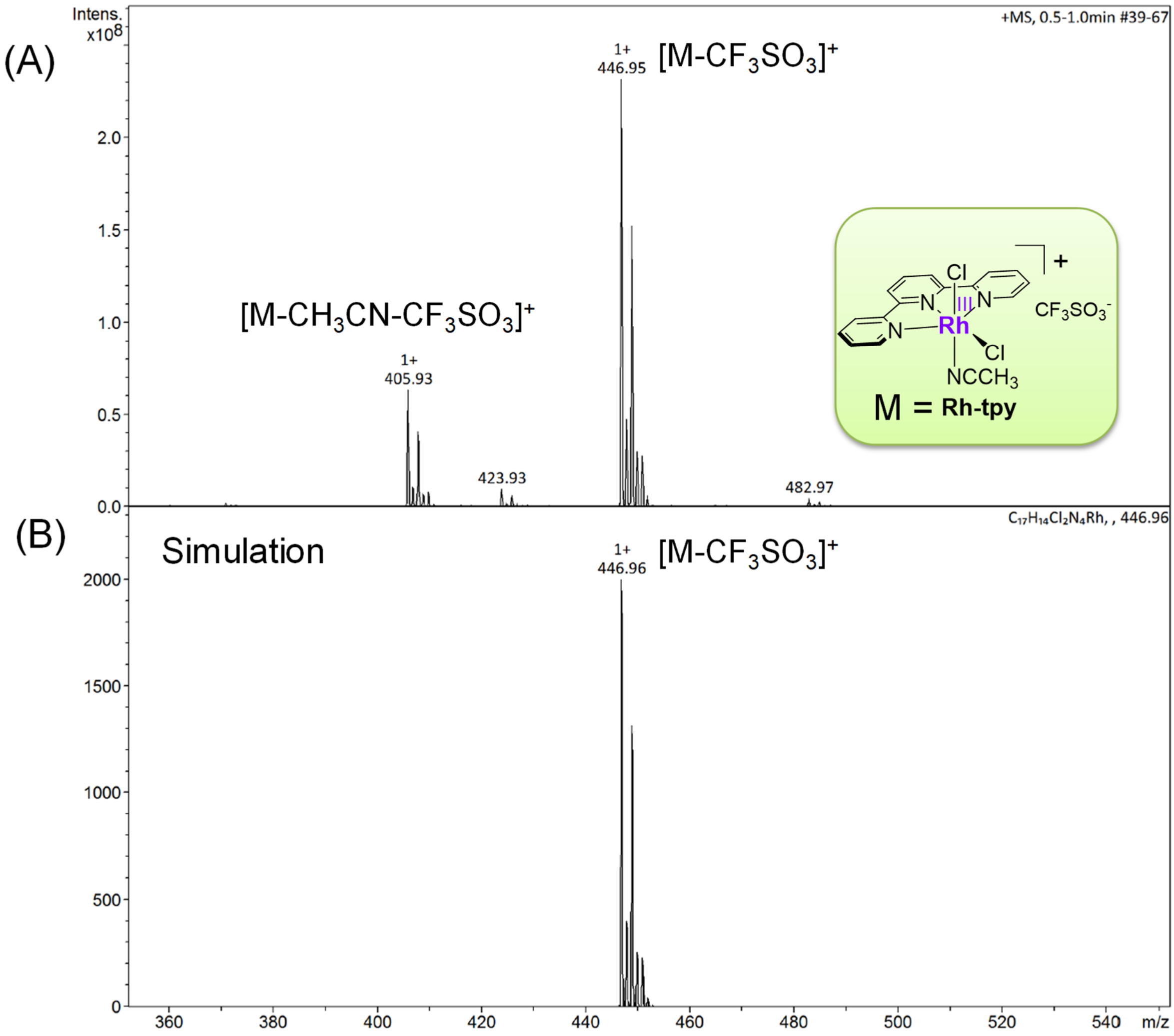

Figure S8. Experimental positive ESI-MS spectrum of $[Rh(tpy)(CH_3CN)Cl_2](CF_3SO_3)$ (A) and simulation of the main isotopic massif at *m/z* = 446.96 corresponding to $[Rh(tpy)(CH_3CN)Cl_2]^+$ (B).

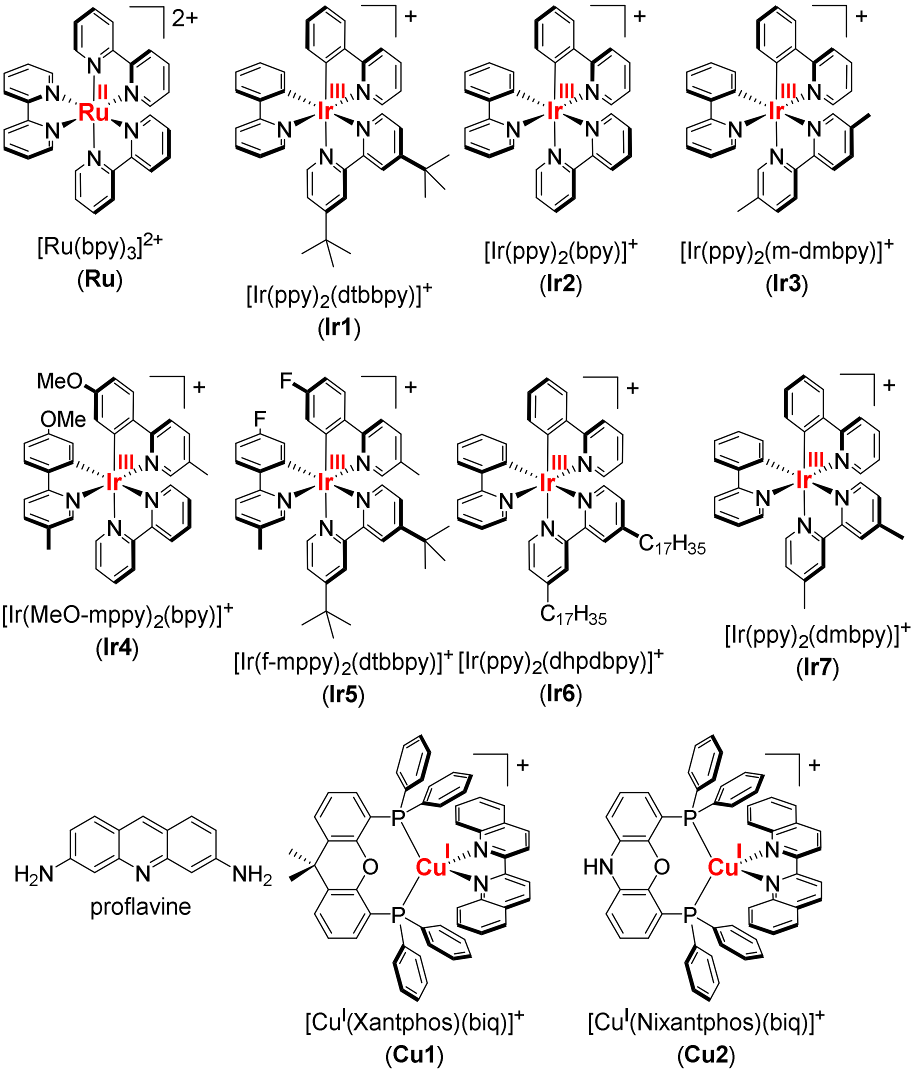

Scheme S1. Photosensitizers previously reported in three-component systems for light-driven H_2 production using Rh catalysts in aqueous or hydro-organic solutions.

Table S8. Summary of the experimental conditions and photocatalytic performances of the various three-component systems (SD/PS/Cat) using Rh catalysts in hydro-organic solution.

PS conc.	Cat conc.	PS/Cat Ratio	SD conc.	Solvant	Irrad. Source (λ)	TON $_{Cat}$	V_{H2} in mL (hours)	Vol. of solution	Author (Year)
**$[Ir(ppy)_2$ (m-dmbpy)]$^+$ (Ir3)** 100 µM	**$[Rh(bpy)_3]^{3+}$ (Rh1)** 100 µM	1/1	TEOA 0.6 M	MeCN/H_2O 9/1	Blue LED (460 nm)	70	1.72 (20)	10 mL	Bernhard (2008) [12]
**[Ir(MeO-mppy)$_2$ (bpy)]$^+$(Ir4)** 100 µM	**$[Rh(dfbpy)_3]^{3+}$ (Rh12)** 100 µM	1/1	TEOA 0.6 M	MeCN/H_2O 9/1	Blue LED (460 nm)	60	1.47 (20)	10 mL	
**Ir3** 100 µM	**$[Rh(dMeObpy)_3]^{3+}$ (Rh13)** 100 µM	1/1	TEOA 0.6 M	MeCN/H_2O 9/1	Blue LED (460 nm)	40	0.98 (20)	10 mL	
**[Ir(f-mppy)$_2$ (dtbbpy)]$^+$ (Ir5)** 100 µM	**$[Rh(dtbbpy)_3]^{3+}$ (Rh11)** 100 µM	1/1	TEOA 0.6 M	MeCN/H_2O 9/1	Blue LED (460 nm)	150	3.68 (20)	10 mL	
**Ir5** 150 µM	**Rh11** 50 µM	3/1	TEA 0.6 M	THF/H_2O 8/2	Blue LED (460 nm)	5196	63.7 (20)	10 mL	
**$[Ir(ppy)_2$ (dhpdbpy)]$^+$ (Ir6)** 1 mM	**$[Rh(dhpdbpy)_2$ $Cl_2]^+$ (Rh14)** 62.5 µM	16/1	Oxalic Acid 75 mM	tol/H_2O/ Oct-OH/ Oct-NH_2 3.8/4/0.2/1	Blue LED (460 nm)	400	5.51 (17)	9 mL	Bernhard (2017) [13]
**$[Ir(ppy)_2(bpy)]^+$ (Ir2)** 500 µM	**[Rh_2(µ-OAc)$_4$ $(H_2O)_2$] (Rh5)** 5 µM	100/1	TEA 0.36 M	THF/H_2O 68/32	Xe Lamp (>380 nm)	3857	9.45 (12)	10 mL	Kataoka (2019) [14]
	**[Rh_2(µ-CF_3CO_2)$_4$ $(H_2O)_2$] (Rh15)** 5 µM	100/1	TEA 0.36 M	THF/H_2O 68/32	Xe Lamp (>380 nm)	3334	8.18 (12)	10 mL	
	**[Rh_2(µ-CCl_3CO_2)$_4$ $(H_2O)_2$] (Rh16)** 5 µM	100/1	TEA 0.36 M	THF/H_2O 68/32	Xe Lamp (>380 nm)	3138	7.69 (12)	10 mL	
	**Rh11** 10 µM	50/1	TEA 0.36 M	THF/H_2O 68/32	Xe Lamp (>380 nm)	2362	5.79 (12)	10 mL	
**$[Ir(ppy)_2(dmbpy)]^+$ (Ir7)** 500 µM	**Rh5** 5 µM	100/1	TEA 0.36 M	THF/H_2O 68/32	Xe Lamp (>380 nm)	~6500	~15.9 (12)	10 mL	Kataoka (2019) [15]
**$[Ir(ppy)_2(dtbbpy)]^+$ (Ir1)** 500 µM	**Rh5** 5 µM	100/1	TEA 0.36 M	THF/H_2O 68/32	Xe Lamp (>380 nm)	9886	24.2 (12)	10 mL	
**Ir1** 50 µM	**[Rh_2(µ-OAc)$_2$ $(OAc)_2$] (Rh17)** 50 µM	1/1	TEA 0.6 M	THF/H_2O 8/2	Artificial sun (>350 nm)	16	0.20 (20)	10 mL	Wang (2012) [16]
	**[$Rh_2(bpy)_2$ (µ-OAc)$_4$$(OAc)_2$] (Rh8)** 50 µM	1/1	TEA 0.6 M	THF/H_2O 8/2	Artificial sun (>350 nm)	62	0.76 (20)	10 mL	
	**[$Rh_2(phen)_2$ (µ-OAc)$_2$$(OAc)_2$] (Rh9)** 50 µM	1/1	TEA 0.6 M	THF/H_2O 8/2	Artificial sun (>350 nm)	379	4.64 (20)	10 mL	
**Ir2** 926 µM	**$[Rh(py)_4Cl_2]^+$ (Rh18)** 926 µM	1/1	TEOA 0.56 M	MeCN/H_2O 9/1	LED (450 nm)	~15	~7.35 (3)	21.6 mL	Kozlova (2016) [17]
	**$[Rh(4\text{-pic})_4Cl_2]^+$ (Rh19)** 926 µM	1/1	TEOA 0.56 M	MeCN/H_2O 9/1	LED (450 nm)	~16	~7.84 (3)	21.6 mL	
	**Rh19[a]** 926 µM	1/1	TEOA 0.56 M	MeCN/H_2O 9/1	LED (450 nm)	100	49.0 (5.5)	21.6 mL	
	**$[Rh(3\text{-pic})_4Cl_2]^+$ (Rh20)** 926 µM	1/1	TEOA 0.56 M	MeCN/H_2O 9/1	LED (450 nm)	~18	~8.82 (3)	21.6 mL	
	**[Rh(4-pyCOOH)$_3$ (4-pyCOO)Cl_2]$^+$ (Rh21)** 926 µM	1/1	TEOA 0.56 M	MeCN/H_2O 9/1	LED (450 nm)	~18.5	~9.07 (3)	21.6 mL	
**Ir2** 926 µM	**[RhCl$(H_2O)_2$ $(py)_3]^+$ (Rh22)** 926 µM	1/1	TEOA 0.56 M	MeCN/H_2O 9/1	LED (450 nm)	~15	~7.35 (1)	21.6 mL	Vasilchenko& Kozlova (2018) [18]
**[Cu(Xantphos) (biq)]+ (Cu1)** 360 µM	**$[Rh(dmbpy)_2Cl_2]^+$ (Rh2)** 1 µM	360/1	DMA 1.57 M	DMF/H_2O 96/4	Blue LED (447.5 nm)	140	0.017 (1)	5.1 mL	White (2018) [19]

	**Rh2**<br>5 µM	72/1	DMA<br>1.57 M	DMF/H_2O<br>96/4	Blue LED<br>(447.5 nm)	72	0.044<br>(1)	5.1 mL	
	**Rh2**<br>10 µM	36/1	DMA<br>1.57 M	DMF/H_2O<br>96/4	Blue LED<br>(447.5 nm)	53	0.066<br>(1)	5.1 mL	
	**Rh2**<br>36 µM	10/1	DMA<br>1.57 M	DMF/H_2O<br>96/4	Blue LED<br>(447.5 nm)	25	0.11<br>(1)	5.1 mL	
	**$[Rh(bpy)_2Cl_2]^+$**<br>**(Rh23)**<br>36 µM	10/1	DMA<br>1.57 M	DMF/H_2O<br>96/4	Blue LED<br>(447.5 nm)	22	0.10<br>(1)	5.1 mL	
	**$[Rh(dmebpy)_2Cl_2]^+$**<br>**(Rh24)**<br>36 µM	10/1	DMA<br>1.57 M	DMF/H_2O<br>96/4	Blue LED<br>(447.5 nm)	43	0.19<br>(1)	5.1 mL	
**Cu1**<br>360 µM	**Rh2**<br>36 µM	10/1	DMA<br>1.55 M	DMF/H_2O<br>96/4	Blue LED<br>(447.5 nm)	260	1.18<br>(3)	5.1 mL	White<br>(2019) [20]
**[Cu(Nixantphos)**<br>**(biq)]+ (Cu2)**<br>360 µM	**Rh2**<br>36 µM	10/1	DMA<br>1.55 M	DMF/H_2O<br>96/4	Blue LED<br>(447.5 nm)	180	0.81<br>(3)	5.1 mL	
**Cu1**<br>360 µM	**Rh2**<br>36 µM	10/1	DMA<br>0.8 M	DMF/H_2O<br>96/4	Blue LED<br>(447.5 nm)	151	0.69<br>(3)	5.1 mL	White<br>(2020) [21]
	**Rh2**<br>36 µM	10/1	*p*-DMT<br>0.8 M	DMF/H_2O<br>96/4	Blue LED<br>(447.5 nm)	197	0.88<br>(3)	5.1 mL	

TON_{Cat} and TOF_{Cat} are respectively the maximum turnover number and the initial turnover frequencies ($TOF_{Cat} = TON_{Cat}\ h^{-1}$) per catalyst exhibited by the system. V_{H2} is the total volume of H_2 produced after a given time of irradiation. n.d. : non determined. bpy is 2,2'-bipyridine, dmbpy is 4,4'-dimethyl-2,2'-bipyridine, m-dmbpy is 5,5'-dimethyl-2,2'-bipyridine, dtbbpy is 4,4′-di-tertbutyl-2,2′-bipyridine, dfbpy is 5,5'-difluoro-2,2'-bipyridine, dMeObpy is 5,5'-dimethoxy-2,2'-bipyridine, ppy is 2-phenylpyridine, MeO-mppy is 5-Methyl-2-(4-methoxyphenyl)pyridine, f-mppy is 5-methyl-2-(4-fluorophenyl)pyridine, dhpdbpy is 4,4′-diheptadecyl-2,2′-bipyridine, dmebpy is 2,2′-bipyridine-4,4′-dimethyl ester, py is pyridine, 4-pic is 4-picoline, 3-pic is 3-picoline, 4-pyCOOH is pyridine-4-carboxylic acid, Xantphos is 4,5-bis(diphenylphosphino)-9,9-dimethylxanthene, Nixantphos is 4,6-bis-(diphenylphosphino)-10H-phenoxazine, biq is 2,2′-biquinoline, OAc is acetate, phen is phenanthroline, tol is toluene, oct-OH is 1-octanol, Oct-NH_2 is 1-octylamine, THF is tetrahydrofuran, DMF is N,N-diethylformamide, TEOA is triethanolamine, TEA is triethylamine, DMA is N,N-dimethylaniline, *p*-DMT is N,N-dimethyl-*p*-toluidine.

[a] Addition of 4-picoline (2 M).

Table S9. Summary of the experimental conditions and photocatalytic performances of various three-component systems (SD/PS/Cat) using Rh catalysts in pure aqueous solution.

PS conc.	Cat conc.	PS/Cat Ratio	SD conc.	pH	Irrad. Source (λ)	TON_{Cat}	V_{H2} in mL (hours)	Vol. of solution	Author (Year)
**$[Ru(bpy)_3]^{2+}$(Ru)** 440 µM	**$[Rh(bpy)_3]^{3+}$ (Rh1)**[a] 1.56 mM	1/3.5	EDTA 0.2 M	5.2	halogen Lamp (>400 nm)	6	1.6 (3)	7 mL	Lehn & Sauvage (1979) [22]
**Ru** 190 µM	**$[RhCl(dpm)_3]^{3-}$ (Rh4)** 190 µM	1/1	H_2A 0.1 M	5.0	Projector Lamp (>408 nm)	1000	18.6 (1.55)	4 mL	Oishi (1987) [23]
**Ru** 667 µM	**Rh4** 167 µM	4/1	H_2A 16.7 mM	4.5	halogen W-Lamp (>400 nm)	>2000	>24.5 (4)	3 mL	Bauer (1994) [24]
**Ru** 40 µM	**$[Rh_2(\mu\text{-}OAc)_4(H_2O)_2]$ (Rh5)**[b] 0.1 mM	1/2.5	EDTA 30 mM	5.0[c]	Xe Lamp (240-550 nm)	4.0	0.178 (6)	10 mL	Sakai (2010) [25]
	**$[Rh_2(\mu\text{-}gly)_4(H_2O)_2]$ (Rh6)**[b] 0.1 mM	1/2.5	EDTA 30 mM	5.0[c]	Xe Lamp (240-550 nm)	4.5	0.201 (6)	10 mL	
	**$[Rh_2(\mu\text{-}CF_3CO_2)_4(acetone)_2]$ (Rh7)**[b] 0.1 mM	1/2.5	EDTA 30 mM	5.0[c]	Xe Lamp (240-550 nm)	2.3	0.104 (6)	10 mL	
	**$[Rh_2(bpy)_2(\mu\text{-}OAc)_2(OAc)_2]$ (Rh8)**[b] 0.1 mM	1/2.5	EDTA 30 mM	5.0[c]	Xe Lamp (240-550 nm)	1.9	0.086 (6)	10 mL	
	**$[Rh_2(\mu\text{-}BL)_4]^{2+}$ (Rh10)**[b] 0.1 mM	1/2.5	EDTA 30 mM	5.0[c]	Xe Lamp (240-550 nm)	0.09	0.004 (6)	10 mL	
**$[Ir(ppy)_2(dtbbpy)]^+$ (Ir1)** 50 µM	**$[Rh_2(phen)_2(\mu\text{-}OAc)_2(OAc)_2]$ (Rh9)** 50 µM	1/1	TEA 0.6 M	n.d.	Artificial sun (>350 nm)	3.3	0.04 (20)	10 mL	Wang (2012) [16]
**Ru** 2 mM	**$[Rh(Cp^*)(bpy)(H_2O)]^{2+}$ (Rh3)** 1 mM	2/1	HA^-/H_2A 1.1 M	3.6	Xe Lamp (>430 nm)	100	4.9 (3)	2 mL	Fukuzumi (2011) [26]
**Ru** 500 µM	**$[Rh(dmbpy)_2Cl_2]^+$ (Rh2)** 10 µM	50/1	HA^-/H_2A 1.1 M	4.0	Xe Lamp (400-700 nm)	640[g]	0.78[g] (20)	5 mL	Collomb (2013) [3]
	**Rh2** 5 µM	100/1	HA^-/H_2A 1.1 M	4.0	Xe Lamp (400-700 nm)	865[g]	0.53[g] (20)	5 mL	
	**Rh2** 1 µM	500/1	HA^-/H_2A 1.1 M	4.0	Xe Lamp (400-700 nm)	1010[g]	0.12[g] (8)	5 mL	
**Ru** 100 µM	**Rh2** 10 µM	10/1	HA^-/H_2A 1.1 M	4.0	Xe Lamp (400-700 nm)	320	0.39 (5)	5 mL	
	**Rh4** 10 µM	10/1	HA^-/H_2A 1.1 M	5.0	Xe Lamp (400-700 nm)	57	0.07 (13.5)	5 mL	
	**Rh3** 10 µM	10/1	HA^-/H_2A 1.1 M	3.6	Xe Lamp (400-700 nm)	108	0.13 (7)	5 mL	
**$[Ir(ppy)_2(bpy)]^+$ (Ir2)** 100 µM	**Rh2** 10 µM	10/1	HA^-/H_2A 1.1 M	4.0	Xe Lamp (400-700 nm)	170	0.21 (12)	5 mL	
**Ru** 530 µM	**Rh2** 10 µM	53/1	HA^-/H_2A 0.1 M	4.5[d]	Xe Lamp (400-700 nm)	1139[g]	1.40[e] (21.7)	5 mL	This work
	**Rh2** 5 µM	106/1	HA^-/H_2A 0.1 M	4.5[d]	Xe Lamp (400-700 nm)	778[g]	0.48[e] (24)	5 mL	
	**Rh2** 1 µM	530/1	HA^-/H_2A 0.1 M	4.5[d]	Xe Lamp (400-700 nm)	772[g]	0.09[e] (22)	5 mL	
	**$[Rh(tpy)(CH_3CN)Cl_2]^+$ (Rhtpy)** 10 µM	53/1	HA^-/H_2A 0.1 M	4.5[d]	Xe Lamp (400-700 nm)	300[g]	0.37[e] (21.5)	5 mL	
	**Rhtpy** 5 µM	106/1	HA^-/H_2A 0.1 M	4.5[d]	Xe Lamp (400-700 nm)	558[g]	0.34[e] (25)	5 mL	
	**Rhtpy** 1 µM	530/1	HA^-/H_2A 0.1 M	4.5[d]	Xe Lamp (400-700 nm)	1012[g]	0.12[e] (22.3)	5 mL	

TON_{Cat} is the corrected value of maximum turnover number per catalyst exhibited by the system. V_{H2} is the total volume of H_2 produced after a given time of irradiation. n.d. : non determined. bpy is 2,2'-bipyridine, dmbpy is 4,4'-dimethyl-2,2'-bipyridine, dtbbpy is 4,4′-di-tertbutyl-2,2′-bipyridine, ppy is 2-phenylpyridine, dpm is

diphenylphosphinobenzen-*m*-sulphonate, Cp* is pentamethylcyclopentadiene, tpy is 2,2':6',2''-terpyridine, OAc is acetate, gly is glycolate, BL is 1,3-diisocyanopropane, phen is phenanthroline, TEOA is triethanolamine, EDTA is , HA^- and H_2A are respectively ascorbate and ascordic acid.
[a] Photocatalysis at 30°C ; [b] addition of methylviologen nitrate (2 mM) ; [c] use of an acetate buffer (0.1 M) ; [d] use of an acetate buffer (1 M) ; [e] The values of TON_{Cat}* and V_{H2}* has been corrected by subtracting the production of H_2 stemming from **Ru** without catalyst.

References

1. Dolomanov, O. V.; Bourhis, L. J.; Gildea, R. J.; Howard, J. A. K.; Puschmann, H., OLEX2: a complete structure solution, refinement and analysis program. *Journal of Applied Crystallography* **2009,** 42, 339-341.
2. Pavlishchuk, V. V.; Addison, A. W., Conversion constants for redox potentials measured versus different reference electrodes in acetonitrile solutions at 25°C. *Inorg. Chim. Acta.* **2000,** 298, 97-102.
3. Stoll, T.; Gennari, M.; Serrano, I.; Fortage, J.; Chauvin, J.; Odobel, F.; Rebarz, M.; Poizat, O.; Sliwa, M.; Deronzier, A.; Collomb, M. N., $[Rh^{III}(dmbpy)_2Cl_2]^+$ as a Highly Efficient Catalyst for Visible-Light-Driven Hydrogen Production in Pure Water: Comparison with Other Rhodium Catalysts. *Chem.-Eur. J.* **2013,** 19, 782-792.
4. Baffert, C.; Dumas, S.; Chauvin, J.; Leprêtre, J.-C.; Collomb, M.-N.; Deronzier, A., Photoinduced oxidation of $[Mn(L)_3]^{2+}$ and $[Mn_2O_2(L)_4]^{3+}$ (L = 2,2'-bipyridine and 4,4'-dimethyl-2,2'-bipyridine) with the $[Ru(bpy)_3]^{2+}$/-aryl diazonium salt system. *PhysChemChemPhys* **2005,** 7, 202-210.
5. Juris, A.; Balzani, V.; Barigelletti, F.; Campagna, S.; Belser, P.; Von Zelewsky, A., Ru(II) polypyridine complexes - Photophysics, photochemistry, electrochemistry, and chemi-luminescence. *Coord. Chem. Rev.* **1988,** 84, 85-277.
6. Kirch, M.; Lehn, J.-M.; Sauvage, J.-P., Hydrogen Generation by Visible Light Irradiation of Aqueous Solutions of Metal Complexes. An approach to the photochemical conversion and storage of solar energy. *Helv. Chim. Acta* **1979,** 62, 1345-1384.
7. Sivanesan, A.; John, S. A., Determination Of L-dopa using electropolymerized 3,3 ',3 '',3 '''-tetraaminophthalocyanatonickel (II) film on glassy carbon electrode. *Biosensors & Bioelectronics* **2007,** 23, 708-713.
8. Varma, S.; Castillo, C. E.; Stoll, T.; Fortage, J.; Blackman, A. G.; Molton, F.; Deronzier, A.; Collomb, M.-N., Efficient Photocatalytic Hydrogen Production in Water Using a Cobalt(III) Tetraaza-Macrocyclic Catalyst: Electrochemical Generation of the Low-Valent Co(I) Species and its Reactivity Toward Proton Reduction. *PhysChemChemPhys* **2013,** 15, 17544-17552.
9. Baxendale, J. H.; Fiti, M., Transient species in the reactions of some pyridyl complex ions with hydrated electrons. *J. Chem. Soc., Dalton Trans.* **1972**, 1995-1998.
10. Gueret, R.; Castillo, C. E.; Rebarz, M.; Thomas, F.; Hargrove, A.-A.; Pécaut, J.; Sliwa, M.; Fortage, J.; Collomb, M.-N., Cobalt(III) tetraaza-macrocyclic complexes as efficient catalyst for photoinduced hydrogen production in water: theoretical investigation of the electronic structure of the reduced species and mechanistic insight. *J. Photochem. Photobiol. B: Biol.* **2015,** 152, 82-94.
11. Müller, P.; Brettel, K., $[Ru(bpy)_3]^{2+}$ as a reference in transient absorption spectroscopy: differential absorption coefficients for formation of the long-lived ^{3}MLCT excited state. *Photochem. Photobiol. Sci.* **2012,** 11, 632-636.

12. Cline, E. D.; Adamson, S. E.; Bernhard, S., Homogeneous Catalytic System for Photoinduced Hydrogen Production Utilizing Iridium and Rhodium Complexes. *Inorg. Chem.* **2008,** 47, 10378-10388.
13. Kagalwala, H. N.; Chirdon, D. N.; Mills, I. N.; Budwal, N.; Bernhard, S., Light-Driven Hydrogen Generation from Microemulsions Using Metallosurfactant Catalysts and Oxalic Acid. *Inorg. Chem.* **2017,** 56, 10162-10171.
14. Kataoka, Y.; Yano, N.; Kohara, Y.; Tsuji, T.; Inoue, S.; Kawamoto, T., Experimental and Theoretical Study of Photochemical Hydrogen Evolution Catalyzed by Paddlewheel-Type Dirhodium Complexes with Electron Withdrawing Carboxylate Ligands. *ChemCatChem* **2019,** 11, 6218-6226.
15. Kataoka, Y.; Yano, N.; Handa, M.; Kawamoto, T., Intrinsic hydrogen evolution capability and a theoretically supported reaction mechanism of a paddlewheel-type dirhodium complex. *Dalton Trans.* **2019,** 48, 7302-7312.
16. Xie, J.; Li, C.; Zhou, Q.; Wang, W.; Hou, Y.; Zhang, B.; Wang, X., Large Improvement in the Catalytic Activity Due to Small Changes in the Diimine Ligands: New Mechanistic Insight into the Dirhodium(II,II) Complex-Based Photocatalytic H_2 Production. *Inorg. Chem.* **2012,** 51, 6376-6384.
17. Vasilchenko, D. B.; Tkachev, S. V.; Kurenkova, A. Y.; Kozlova, E. A.; Kozlov, D. V., Photocatalytic hydrogen evolution by iridium(III)–rhodium(III) system: Effect of the free ligand addition. *International Journal of Hydrogen Energy* **2016,** 41, 2592-2597.
18. Vasilchenko, D.; Tkachev, S.; Baidina, I.; Korolkov, I.; Berdyugin, S.; Kozlova, E.; Kozlov, D., Preparation of a rhodium(iii) cis-diaquacomplex by protic acid induced oxalate-release from mer-[Rh(C2O4)Cl(py)3]. *New Journal of Chemistry* **2018,** 42, 19637-19643.
19. McCullough, B. J.; Neyhouse, B. J.; Schrage, B. R.; Reed, D. T.; Osinski, A. J.; Ziegler, C. J.; White, T. A., Visible-Light-Driven Photosystems Using Heteroleptic Cu(I) Photosensitizers and Rh(III) Catalysts To Produce H_2. *Inorg. Chem.* **2018,** 57, 2865-2875.
20. Saeedi, S.; Xue, C.; McCullough, B. J.; Roe, S. E.; Neyhouse, B. J.; White, T. A., Probing the Diphosphine Ligand's Impact within Heteroleptic, Visible-Light-Absorbing Cu(I) Photosensitizers for Solar Fuels Production. *ACS Appl. Energy Mater.* **2019,** 2, 131-143.
21. Saeedi, S.; White, T. A., Insight into the Reductive Quenching of a Heteroleptic Cu(I) Photosensitizer for Photocatalytic H_2 Production. *ACS Appl. Energy Mater.* **2020,** 3, 56-65.
22. Kirch, M.; Lehn, J. M.; Sauvage, J. P., Hydrogen Generation by Visible-Light Irradiation of Aqueous-Solutions of Metal-Complexes - Approach to the Photo-Chemical Conversion and Storage of Solar-Energy. *Helv. Chim. Acta* **1979,** 62, 1345-1384.
23. Oishi, S., A Water-Soluble Wilkinsons Complex as Homogeneous Catalyst for the Photochemical Reduction of Water. *J. Mol. Catal.* **1987,** 39, 225-232.
24. Bauer, R.; Werner, H. A. F., Investigations on a Homogeneous Wilkinsons Catalyst for the Water Photolysis. *Int. J. Hydrogen Energy* **1994,** 19, 497-499.
25. Tanaka, S.; Masaoka, S.; Yamauchi, K.; Annaka, M.; Sakai, K., Photochemical and thermal hydrogen production from water catalyzed by carboxylate-bridged dirhodium(II) complexes. *Dalton Trans.* **2010,** 39, 11218-11226.
26. Fukuzumi, S.; Kobayashi, T.; Suenobu, T., Photocatalytic Production of Hydrogen by Disproportionation of One-Electron-Reduced Rhodium and Iridium–Ruthenium Complexes in Water. *Angew. Chem. Int. Ed.* **2011,** 50, 728-731.
